# Diffusion Tensor Imaging in Parkinson's Disease and Parkinsonian Syndrome: A Systematic Review

**DOI:** 10.3389/fneur.2020.531993

**Published:** 2020-09-25

**Authors:** Yu Zhang, Marc A. Burock

**Affiliations:** ^1^Department of Psychiatry, War Related Illness and Injury Study Center, Veterans Affairs Palo Alto Health Care System, Palo Alto, CA, United States; ^2^Department of Psychiatry, Mainline Health, Bryn Mawr Hospital, Bryn Mawr, PA, United States

**Keywords:** diffusion tensor imaging (DTI), fractional anisotropy (FA), Parkinson's progression marker initiative (PPMI), diffusion tensor tractography (DTT), dopaminergic pathway, substantia nigra (SN), Parkinsion's disease (PD)

## Abstract

Diffusion tensor imaging (DTI) allows measuring fractional anisotropy and similar microstructural indices of the brain white matter. Lower than normal fractional anisotropy as well as higher than normal diffusivity is associated with loss of microstructural integrity and neurodegeneration. Previous DTI studies in Parkinson's disease (PD) have demonstrated abnormal fractional anisotropy in multiple white matter regions, particularly in the dopaminergic nuclei and dopaminergic pathways. However, DTI is not considered a diagnostic marker for the earliest Parkinson's disease since anisotropic alterations present a temporally divergent pattern during the earliest Parkinson's course. This article reviews a majority of clinically employed DTI studies in PD, and it aims to prove the utilities of DTI as a marker of diagnosing PD, correlating clinical symptomatology, tracking disease progression, and treatment effects. To address the challenge of DTI being a diagnostic marker for early PD, this article also provides a comparison of the results from a longitudinal, early stage, multicenter clinical cohort of Parkinson's research with previous publications. This review provides evidences of DTI as a promising marker for monitoring PD progression and classifying atypical PD types, and it also interprets the possible pathophysiologic processes under the complex pattern of fractional anisotropic changes in the first few years of PD. Recent technical advantages, limitations, and further research strategies of clinical DTI in PD are additionally discussed.

## Introduction

Parkinson's disease (PD) is the second most common neurodegenerative disorder after Alzheimer disease. PD generally begins with motor symptoms, including bradykinesia, rigidity, resting tremor, and postural instability. PD also includes numerous non-motor symptoms (such as cognitive impairment, depression, sleep behavioral problems, and olfactory dysfunction) (1). The classical neuropathologic hallmark of PD involves the loss of dopaminergic neurons in the substantia nigra (SN), resulting in a decreased dopaminergic output through the cortico-basal ganglia-thalamocortical motor circuit, which causes dysregulation of motor functions (2). Another neuropathologic characteristic of PD is the presence of Lewy bodies (3) and neurites in both neuron bodies and axons, including aggregates of the α-synuclein protein (4). When PD begins, the complex features of motor and non-motor symptoms reflect the progression of underlying pathologies, including α-synuclein immunoreactive inclusions in Lewy bodies; loss of dopaminergic, cholinergic, serotonergic, and noradrenergic projections from the brainstem to the midbrain and basal forebrain; and, finally, to the neocortex (5). Over the course of PD, patients commonly experienced comorbid neuropsychiatric disturbances, including depression, behavioral and cognitive deficits, and dementia, which need to be differentiated from other neurodegenerative causes such as dementia with Lewy bodies (DLB) and Alzheimer's disease. On the other hand, the term “parkinsonism” refers to motor syndromes that also present in PD, including bradykinesia, cogwheel rigidity, resting tremor, a slow shuffling gait, and imbalance. The atypical causes of parkinsonism that mimic idiopathic PD include multiple system atrophy (MSA), progressive supranuclear palsy (PSP), or corticobasal syndrome (CBS), as well as drug-induced parkinsonism and others. The most common motor and non-motor syndromes of PD, and other causes of parkinsonism that are involved in PD differential diagnosis, are listed in Table 1.

**Table 1:** Motor and non-motor syndromes of PD and other causes of Parkinsonism.

**Idiopathic PD motor syndromes**	**Diseases showing similar motor dysfunctions**	**Idiopathic PD non-motor syndromes**	**Diseases showing similar non-motor dysfunctions**
- Resting tremor - Bradykinesia - Rigidity - Postural instability - Freezing of gait - Micrographia - Decreased facial movements - Unwanted rapid movements - Other: dystonia, speech problem, Difficulty swallowing, Sexual Dysfunction, Cramping	- Multiple system atrophy (MSA) - Progressive supranuclear palsy (PSP) - Corticobasal syndrome (CBS) - Other: Essential Tremor (ET), drug-induced parkinsonism, normal -pressure hydrocephalus	- Neurodegeneration: Mild cognitive impairment, executive dysfunction, Dementia - Mood and emotion: depression, anxiety, apathy - Smell: hyposmia - Sleep: REM behavioral disorder, Insomnia, Excessive daytime sleepiness - Hallucinations, delusions - Other: fatigue, Autonomic, Pain, Skin problems, Impulsive behaviors due to the side effects of medication	- Dementia with Lewy bodies (DLB) - Demented PD (PDD) vs. Alzheimer's disease - PD with depression

In PD, the loss of dopaminergic neurons and the accumulation of Lewy bodies are typically accompanied by damage of neuroglial cells and demyelination of axons with increasing microglia concentration in extracellular spaces. It is therefore plausible that the detection of extracellular microstructural abnormalities in brain regions with dopaminergic neurons and along dopaminergic pathways might be a biomarker of incipient PD.

Diffusion Tensor Imaging (DTI), one of the magnetic resonance imaging (MRI) sequences, has been employed to measure white matter microstructural integrity in neurodegenerative diseases, as well as to visualize brain fiber connections via tractography (6). Many quantitative DTI indices (as summarized in Table 2) could be derived from a clinical DTI sequence. Fractional anisotropy (FA), radial (RD), axial (AD), and mean diffusivity (MD) have been most commonly used to describe the degree of random motion of water molecules on a microscopic scale. Specifically, FA, which measures the directionality of random water motion, has been used to probe nerve fiber arrangements, axonal integrity, and the degree of axonal myelination (64). FA is clinically feasible to deduce the microstructural integrity of brain tissues, especially preferred to oriented tissues, such as white matter and fiber-tract architectures. MD measures the magnitude of water diffusion. A high MD is thought to indicate broad cellular damages including edema and necrosis. MD is used clinically to capture these microstructural alterations in both gray and white matter tissues (65). AD measures the magnitude of diffusion along the main axis, and RD measures the magnitude of transverse diffusion. In animal studies, increased RD appears to describe myelin pathology-induced myelin thinning (66), while decreased AD indicates acute axonal injury but does not correlate with chronic axonal damage (67). However, the clinical utilities of these DTI metrics have to consider several limitations: (1) All these DTI metrics lack validations with specific neuropathology in postmortem brain. (2) The interpretation of RD/AD might be difficult in voxels containing isotropic structure. Wheeler-Kingshott and Cercignani (68) reported that fictitious changes between RD and AD could occur in areas of low anisotropy, severe pathology, partial volume, and crossing-fibers. They advised that interpretations of RD/AD should be strongly discouraged under such circumstances. (3) There is no appropriate neurobiological implication for AD increases. Linking the increased AD to axonal recovery (as a reverse response of axonal injury) remains questioned, because many studies found AD increase is associated with neurodegenerative disorders such as dementia (69). Despite these limitations, the decreased FA and increased MD have been found correlated with neuronal degeneration (70) and degeneration caused by dopamine loss (71). Further, previous studies (72, 73) reported that abnormal FA values are detected in PD prior to atrophy, suggesting the usefulness of DTI measures as a biomarker of PD in clinical studies.

**Table 2:** Summary 1of common microstructural and connectivity indices derived from DTI, as well as their utilities, interpretations, and clinical correlations in PD.

**Measures**	**Modality**	**Analysis**	**Interpretation of abnormal indicates**	**Clinical correlation in PD**
**Microstructural indices**				
Fractional anisotropy (FA)	Diffusion tensor imaging	All analytic types	Low FA: reduced integrity, axonal loss, demyelination, etc. High FA: improved axonal alignment, re-myelination, crossing-fiber, etc.	- Low FA of the SN (7–12), NST (13), thalamic tract (anterior nucleus) (14) correlated with motor dysfunction (UPDRS-III). - Low FA of the basal ganglia regions correlated with non-motor dysfunction (UPDRS except part III) (15). - Low FA of the CC body correlated with the high risk of falls (16). - Low FA of the PPN correlated with severe degree of FOG (17). - Low FA of the entrance to the EC, lateral to the anterior horn of the ventricle, and four ROIs in PFC correlated with severe PIGD symptoms (18). - Low FA correlated of the thalamus with a poor motor speed and balance (19). - Low FA of primarily the frontal and parietal regions correlated with executive, visuospatial dysfunctions (20–24). - Low FA of the parietal regions, CG, UF, ILF SLF, Fornix tracts, correlated with low aggregate cognition, and memory (25, 26). - Low FA of the thalamus (27), left deep temporal cortex (28) correlated with the severe depression. - Low FA of the frontal regions, IFOF correlated with severe sadness (29). - Low FA of the multiple brain tracts, IFL, SFL, cerebellum, IFOF correlated with severe neuroinflammation (30, 31). - Low FA of the Fornix correlated with excessive daytime sleepiness (32). - Low FA of the brainstem correlated with autonomic dysfunction during REM sleep (33). - Low FA of the gyrus rectus correlated with smell loss (34). - Low anisotropy of the CC, Fornix correlated with severe olfactory dysfunction (35). - Low FA of the rostral SN correlated with low DAT-SBR of the putamen (7).
Mean diffusivity (MD)	Diffusion tensor imaging	All analytic types	High MD: atrophy, damaged cellularity, edema, necrosis, etc. less specific to tissue type	- High MD of the SN (36), contralateral Put (37), GP (38), Genu, EC, SCR, ACR (39) corrected with motor dysfunction (UPDRS-III). - High MD of the basal ganglia regions correlated with non-motor dysfunction (UPDRS except part III) (15). - High MD of the CC body correlated with the higher risk of falls (16). - High MD of the PPN (17), PLIC (40) correlated with severe degree of FOG. - High MD of the entrance to the EC, lateral to the anterior horn of the ventricle, and four ROIs in PFC correlated with severe PIGD symptoms (18). - High MD of the brainstem, thalamus, IC and SCR (41), the tracts projecting to the right pre- and primary motor cortices correlated with high tremor scores (42). - High MD of the thalamus, SLF correlated with a worse motor speed and balance (19). - High MD of the frontal, parietal, temporal regions correlated with executive dysfunction (21, 24, 43, 44). - High MD of extensive regions, UF, ILF SLF tracts, correlated with low global cognition dysfunction (26, 45, 46). - High MD of the medial temporal region, Hippocampus, ILF, CG correlated with memory dysfunction (47, 48). - High MD of the IFL, SFL, cerebellum, IFOF correlated with high inflammatory parameters (30). - High MD of the temporal region, ILF, CG, Fornix correlated with language dysfunction (47). - High MD of the brainstem correlated with autonomic dysfunction during REM sleep (33). - High MD of the SN correlated with smell loss (49).
Radial and axial diffusivity (RD, AD)	Diffusion tensor imaging	All analytic types	High RD: de- or dys-myelination, changes in the axonal diameters or density. Low AD: axonal injury. High AD: unclear	- High RD/AD of the SN (7), NST (13), EC, SCR, ACR, IFOF, PTR (39), CST, CG, CC (50) correlated with motor dysfunction (UPDRS-III). - High RD/AD of the basal ganglia regions correlated with non-motor dysfunction (UPDRS except part III) (15). - High RD of the entrance to the EC, lateral to the anterior horn of the ventricle, and four ROIs in PFC correlated with severe PIGD symptoms (18). - High RD of the prefrontal cortex region correlated with executive and visuospatial dysfunction (51). - High RD/AD of the CG associated with global cognitive decline (50). - High RD of the basal forebrain Cholinergic regions correlated with memory and executive dysfunctions (52). - High RD of the IFL, SFL, cerebellum, IFOF correlated with high inflammatory parameters (30). - High RD/AD of the SN correlated with smell loss (49).
Mean kurtosis (MK)	Diffusion kurtosis imaging using multiple *b* values	ROI and whole brain	High MK: more hindered and restricted diffusion environment. Low MK: more tissue complexity	- High MK of the SN correlated with motor dysfunction (H&Y, and UPDRS-III) (53). - High MK of the frontal, temporal, basal ganglia, limbic, and paralimbic regions correlated with motor deficits (UPDRS-III) (54).
Free water (FW)	Reconstructed from DTI, using bi-tensor model	ROIs, local tracts	High FW: increased extra-cellular space can be attributed to atrophy	- High FW of the SN correlated with motor dysfunction (UPDRS-III, H&Y) in overall PD and control groups (55, 56). - High FW of the SN correlated with low cognition (MoCA) in PD and control groups (55, 56). - High FW of the posterior SN correlated with low DAT-SBR of the putamen (55).
**Connectivity indices:**				
Streamline/fiber numbers, density or volume	Diffusion tensor tractography, tensor density imaging (TDI)	Local or whole brain tracts	Low streamline profiles: lost axons, disrupted neuropathway, artifact or crossing-fiber	- Low connectivity matrix of the pallidum–putamen connection correlated with motor dysfunction (UPDRS-III) (57). High connectivity matrix of sensorimotor cortex–putamen correlated with motor dysfunction (UPDRS-III) (57). - Low connectivity metrics between bilateral SMAs correlated with smell loss and motor dysfunction (UPDRS-III) (58). - Low fiber count of the NST correlated with motor dysfunction (UPDRS-III) (59). - Low fiber density of the basal ganglia local connections correlated with motor dysfunction (UPDRS-III) (60).
Intracellular volume fraction (Vic), Orientation dispersion index (OD)	Multiple *b* values fitted to the neurite orientation dispersion and density imaging (NODDI) model	Local tracts	Likely to explain neurite density Low Vic and OD: neuronal loss or loss of neuronal fibers	- Low Vic and OD of the putamen and SNc correlated with increased PD duration and UPDRS-III (61).
Connectome	Graph theory and network measures	Whole brain inter-Connectivity	Likely to explain structural connectivity according to global efficiency, clustering coefficient, path length, etc.	- Low local efficiency between putamen and local regions correlated with motor dysfunction (UPDRS-III) (62). - Low global efficiency and clustering coefficient correlated with decreased CSF levels of a-synuclein, and Aβ_42_ – abnormal aggregation (63).

*SN, substantia nigra; NST, nigrostriatal tract; CC, corpus callosum; PPN, pedunculopontine nucleus; PLIC, posterior limb of the internal capsule; FOG, freezing of gait; PFC, prefrontal cortex; PIGD, postural imbalance and gait difficulty; CG, cingulum, or cingulate tract; UF, uncinate fasciculus; ILF, inferior longitudinal fasciculus; SLF, superior longitudinal fasciculus; IFOF, inferior fronto-occipital fasciculus; Put, putamen; GP, globus pallidum; EC, external capsule; IC, internal capsule; SCR, superior corona radiata; ACR, anterior corona radiata; PTR, posterior thalamic radiation; CST, corticospinal tract; SMA, supplementary motor areas; DAT-SBR, striatal binding ratios on dopamine transporter single photon emission tomography; REM sleep, rapid eye movement sleep*.

This study aims to review a majority of clinically employed DTI studies in investigations of PD, together with a comparison of the results from a longitudinal, early stage, multicenter clinical cohort. It is sought to prove the utilities of DTI as a biomarker of diagnosing PD, correlating clinical symptomatology, and tracking disease progression and treatment effects on PD. Advantages and challenges of clinical application of DTI in PD will be discussed further.

## DTI Assessment of PD Diagnosis

### Non-hypothesis Driven, Voxel-Wise DTI Analyses in PD

The voxel-based whole brain analysis is a computational approach to identify to what extent in brain anatomy there are significant group differences in DTI indices, via a voxel-by-voxel comparison throughout the entire brain. This analysis automatically registers individual DTI maps to a template, which reduces the significant differences in brain anatomy between people. Registration algorithms can be intensity-based, for example, voxel-based analyses (VBA) (74), or tract-based, for example, tract-based spatial statistics (TBSS) (75). The advantages of the whole brain analyses are fully automatic, unbiased, unsupervised, without the need of prior hypotheses. However, the disadvantages of the whole brain approaches could be sensitive to various artifacts and technical issues, which include mis-registration of brain tissues, mis-classification of tissue types, etc. All these matters may confound the statistical analysis and either decrease the sensitivity to true DTI effects or increase the chance of false positives. Multiple studies have utilized non-hypothesis driven DTI analyses (Table 3) in comparing PD with healthy controls (HC). By analyzing datasets with a large variety of sample ages, disease severity, medication status, and non-motor symptoms, these studies reported a heterogeneous, multifocal pattern of abnormal DTI changes. The complex distribution of abnormal DTI changes is consistent with the notion that PD is a multisystem disorder involving several neurotransmitters beyond the loss of only dopamine. A recent review (102) summarized the various anatomical regions where abnormal DTI values correlated with a variety of PD symptomatology, including motor and motor phenotypes as well as non-motor features such as cognitive, mood, olfactory dysfunction, hallucinations/psychosis, and sleep disturbance, such as rapid eye movement sleep behavior disorder (RBD). It is therefore conceivable that the inconsistent findings of DTI abnormality across previous studies reflect the clinical heterogeneity of the study population. In this context, it will be indeed necessary to replicate these findings in a large population, detect imaging-clinical correlations under unique types of dysfunction, and track how these findings may alter during the early course of PD.

**Table 3:** An overview of the non-hypothesis driven DTI studies in Parkinson's disease.

**Author, year**	**Group**	**Age**	**Duration (years)**	**UPDRS-III**	**H&Y**	**Medication**	**Non-motor syndromes**	**Region of FA decrease/MD increase**	**Region of FA increase/MD decrease**
Zhang et al. (76)	25 HC 25 PD	58.4 (9.8) 58.4 (9.3)	5 (2–29)	48.0 (14.0)	1–3	Medicated	w/o factory dysfunction; normal cognition	[FA] bilateral cerebellar hemispheres, right rectus gyrus [MD] bilateral orbitofrontal cortices, bilateral inferior temporal gyri	[FA] *n.s*. [MD] bilateral parietal lobes and left MC
Rae et al. (21)	15 HC 14 PD	64 (50–75) 65 (51–78)	10 (4–20)	20 (14–32)	2.1 (1.5–3)	Medicated (ON/OFF)		[FA] prefrontal and parietal WM, the CC, and the superior CST [MD] prefrontal WM and the CC	
Zhan et al. (11)	20 HC 12 PD	67.4 (8) 67.2 (8)		26.3 (12.2)		Medicatd (OFF)		[FA] superior and inferior MC, superior postcentral gyrus, posterior striatum, frontal WM, and along projections to the SMA, IC, EC, in the proximity of the Put, thalamus, and SN	
Gallagher et al. (22)	15 HC 15 PD	60.3 (10) 62.7 (6.5)	5.6 (5)	10 (12)	1.63	Medicated	w/ executive dysfunction	[FA] ALIC, ACR, body of CC, SS, UF, and deep cerebellar WM [MD] similar and more extensive than distribution of FA	
Kim et al. (77)	64 HC 64 PD	63.0 (8.9) 62.9 (9)	5.3 (5.4)		2 (1-4)	Medicated (OFF)	Normal cognition	[FA] *n.s*. [MD] corticofugal tract, CG, UF, FXST, CC, EC, SLF, PTR, and tracts adjacent to the precuneus and supramarginal gyrus	
Theilmann et al. (23)	26 HC 25 PD	65.9 (8.4) 68.0 (8.9)	7.2 (4.8)	25.4 (8.9)	2.4 (0.3)	Medicated (ON)	w/ impaired cognition	[FA] widespread, bilateral frontal tracts, left parietal and occipital tracts [MD] widespread, bilateral frontal tracts, parietal and occipital tracts	
Auning et al. (51)	19 HC 18 PD	64.6 (6.5) 66.7 (5.1)	2.2 (1.1)	12.3 (6.8)	1.7 (0.6)	Medicated	Slightly depressed	[FA] frontoparietal regions, CC and the posterior CG	
Diez-Cirarda et al. (78)	15 HC 37 PD	65.1 (7.0) 68.0 (6.2)	6.96 (5.6)	21.7 (10.3)	1.9 (0.5)	Medicated (ON)	w/impaired cognition	[FA] right UF [MD] *n.s*.	
Jiang et al. (79)	34 HC 31 PD	69.3 (8) 69.4 (8)	~4 (2)	~20 (9)	~3 (1)	Partly medicated	w/ depression, impaired cognition and self-care ability	[FA] CC, SLF, ILF, CG, optic radiation, left IC and subcortical arcuate fibers	
Koshimori et al. (43)	14 HC 16 PD	67.1 (5.1) 70.5 (5.6)	6.7 (4.2)	25.3 (15.3)		Medicated (ON)	w/ impaired cognition	[FA] *n.s*. [MD] larger area of bilateral frontal and temporal regions and smaller areas of the left parietal and occipital regions	
Skidmore et al. (80)	22 HC 20 PD	64 (9) 61 (13)	5–12	34 (14)	3 (1.5–5)	Medicated (OFF)	w/ impaired cognition, depression		[FA] Rectal gyrus, middle CG, bilateral Put, left thalamus
Vercruysse et al. (81)	15 HC 15 PD	68.1 (6.5) 67.6 (5.6)	7.6 (5.3)	32.5 (9.1)	2.5 (2–2.5)	Medicated	w/o freezing of gait		[FA] body of CC [MD] body of CC, CST, pre-central WM, Anterior cerebellum
Yoo et al. (82)	18 HC 9 PD	54.4 (6.5) 59.6 (8.6)	10.6 (3.9)	14.4 (8.0)	2.2 (0.4)	Medicated (ON)	w/o impulse control disorder	[FA] bilateral orbitofrontal, medial prefrontal, anterior cingulate areas [MD] *n.s*.	
Youn et al. (17)	33 HC 42 PD	69.6 (5.8) 69.1 (6.4)	9.2 (4.0)	20.1 (10.6)	2.3 (0.3)	Medicated (ON)	w/ and w/o freezing of gait	[FA] left thalamus, bilateral orbitofrontal area, bilateral SN [MD] bilateral inferior temporal cortex, orbitofrontal cortex, insula and left frontal area	
Duncan et al. (44)	50 HC 125 PD	65.8 (8.0) 66.0 (10.5)	0.51 (0.4)	26.8 (11)^[b]^	2 (1–3)	Medicated (ON)	w/ impaired cognition	[FA] *n.s*. [MD] forceps minor, CG, SLF, ILF, IFOF, CST, CC and IC	
Price et al. (83)	40 HC 40 PD	68.2 (4.6) 67.8 (5.4)	7.5 (5.1)	2.8 (3.4) 17.6 (10.7)	1.6 (0.8)	Medicated (ON)	w/ some impaired cognition	[FA] genu and body of CC, forceps minor, ATR, IFOF and UF	
Lim et al. (84)	25 HC 14 PD	68.5 (6.6) 69.7 (7.2)	4.4 (3.7)	22.4 (10.6)	1.6 (0.5)	Medicated (OFF)	w/o rapid eye movement sleep behavior disorder	[FA] mainly in both frontal Areas [MD] widespread	
Mole et al. (85)	26 HC 24 PD	64.9 (8.1) 63.4 (10.8)		25 (11)	1.8 (0.4)	Medicated (OFF)		[FA] right UF	[FA] CST, right Put-SMA, bilateral Thalamus-MC
Wang et al. (86)	16 HC 16 PD	68.6 (2.6) 68.9 (6.0)	3.7 (2.9)	20.9 (10.6)		Not mentioned	w/o freezing of gait	[FA] genu and body of CC, left SCR [MD] CC (genu, body, splenium), right SCR, right IC and EC, bilateral PTR, SLF, and corona radiata	
Wen et al. (39)^[a]^	60 HC 54 HY1PD 87 HY2PD	60.3 (10.8) 60.2 (9.6) 62.0 (9.3)	0.5 (0.5) 0.6 (0.6)	14.7 (5.7)^[b]^ 25.1 (8.6)^[*b*]^	1 2	*de-novo*			[FA] CC, forceps minor, the thalamic radiation, ACR, SCR, and PLIC, EC, SLF, ILF, IFOF and CG [MD] aforementioned WM tracts
Canu et al. (87)	28 HC 28 PD	61.9 (8.3) 63.6 (6.5)	9.7 (5.4)	47.3 (8.2)	2.6 (0.5)	Medicated (ON)	w/o impulsive-compulsive behaviors, with depression	[FA] *n.s*. [MD] left pedunculopontine tract and splenium of CC	
Chen et al. (88)	24 HC 18 PD	62.9 (3.7) 62.3 (4.6)	3.1 (2.8)	17.4 (8.7)		Medicated (OFF)		[FA] body of CC, fornix, left hippocampus, and left IFOF	
Chen et al. (24)	33 HC 24 PD	48.6 (7.8) 48.4 (6.5)	3.2 (3.0)	19.1 (13.6)	1.7 (1.0)	Not mentioned	w/ abnormal executive and visuospatial function	[FA] right SLF, right temporal WM, left inferior and superior parietal WM, bilateral ATR, bilateral occipital WM, left IFOF, left CST, left Put [MD] left inferior parietal WM, bilateral occipital WM, right parietal WM, and right posterior SLF	
Chiang et al. (30)	67 HC 66 PD	56.8 (9.8) 58.1 (8.7)	9.4 (4.5)	22.7 (16.8)	2.0 (1.1)	Medicated	w/ inflammation	[FA] left parietal and right occipital ILF, left postcentral and right parietal SLF, left cerebellum, and left IFOF. [MD] left ILF, right SLF, left cerebellum, and left IFOF	
Cousineau et al. (89)^[a]^	179 HC 412 PD		<2		1–2	*de-novo*			[FA] CC, CST, nigro-subthalamo-putaminal-thalamocortical connections
Georgiopoulos et al. (90)	13 HC 22 PD	68 (65–70) 68 (67–70)	7 (2)	20 (16–27)	2 (1.5–3)	Medicated (ON)	w/ olfactory dysfunction		[FA] *n.s*. [MD] left CST, bilateral PLIC, neural tracts adjacent to left SN
Kamagata et al. (54)	28 HC 30 PD	66.5 (10.8) 67.6 (9.8)	6.4 (3.7)	16.1 (8.8)	2.1 (0.9)	Medicated		[FA] left temporal, left limbic, and paralimbic areas [MD] left frontal, left temporal, left limbic, and paralimbic areas	
Lee et al. (91)	30 HC 21 PD	68.6 (6.0) 66.2 (6.8)	7.0 (4.2)	16.4 (5.1)	1.8 (0.5)	medicated	w/o visual hallucination	[FA] the bilateral fronto-temporo-parietal areas, midbrain and pons [MD] *n.s*.	
Luo et al. (41)	26 HC 30 PD-TD	53.4 (10) 54.5 (8)	2.0 (1.7)	25.4 (12)	1.6 (0.5)	Partly medicated (OFF)		[FA] *n.s*. [MD] MCP, SCP, cerebral peduncles, thalamus, IC, and SCR, fornix, ILF, and IFOF	
Chen et al. (92)	30 HC 30 PD	58.0 (9.3) 64.3 (10.3)	5.2 (3.6)	17.9 (9.3)	1.8 (0.9)	Medicated (ON)	No psychiatric, neurological disorder	[FA] olfactory tract, hippocampal CG, SLF (temporal part)	[FA] Corticospinal tract
Guimaraes et al. (50)	137 HC 132 PD	57.8 (9.4) 60.9 (9.8)	7.8 (6.4)	16 (8.2)	2.8 (1.3)	Medicated (ON)		[FA] genu, body, and splenium of CC, IC and EC, corona radiata, PTR, SS, CG and SLF	
Li et al. (93)	22 HC 31 PD	59.7 (8.6) 60.5 (9.3)		26.4 (10.1)	1.6 (0.5)	Not mentioned	w/ depression, memory, olfactory dysfunction	[FA] bilateral ALIC, bilateral EC, right ACR, genu, body and pad of CC, left sagittal layer.	
Minett et al. (94)	48 HC 93 PD	66.0 (7.9) 64.3 (10.8)	0.5 (0.0)	25.9 (1.1)	1.9 (0.1)	Medicated (ON)	Normal cognition	[FA] n.s. [MD] bilateral corona radiata, IC and EC, CC, IFOF, SFOF, forceps minor, CG, SLD, ILF	
Pietracupa et al. (95)	19 HC 16 PD	66.7 (7.7) 69.7 (11)	9.5 (6.2)	29.8 (17)	2.5 (1.1)	Medicated (ON/OFF)	w/o freezing of gait	[FA] *n.s*. [MD] right cingulum (angular bundle)	
Rektor et al. (96)	21 HC 20 PD	57.9 (7.2) 61.9 (7.6)	<5		1–.5	Medicated (ON)	normal cognition	[FA] *n.s*. [MD] left SCR, SLF, EC, IC, temporal, and prefrontal WM	
Taylor et al. (97)^[a]^	45 HC 71 PD	59.6 (11) 61.3 (9)	<2	0.7 (1.6)^[b]^ 21.3 (8.9)^[*b*]^		Medicated (OFF)	Normal cognition		[FA] Midbrain, CST, pontine and cerebellar WM, anterior CC, right ACR, Left ILF, IFOF, ACR
Wen et al. (98)^[a]^	61 HC 52 PD-TD	60.2 (10.8) 60.5 (9.6)	0.63(0.7)	0.6 (1.4)^[b]^ 19.8 (9.5)^[*b*]^	1–2	*de-novo*	Normal cognition		[FA] Left ATR, IFOF, bilateral ILF, SLF, SS, right CST [MD] *n.s*.
Guan et al. (99)	46 HC 65 PD	57.8 (9.4) 55.5 (9.5)	4.7 (3.9)	27.1 (14.4)	2.3 (0.7)	Medicated (OFF)		[FA] right UF [MD] right EC and forceps minor, left CG	
This study^[a]^	15 YHC 25 YPD(Y0) 19 YPD(Y3) 60 OHC 104 OPD(Y0) 72 OPD(Y3)	42.3 (6) 46.1 (4) 50.6 (4) 63.4 (8) 63.8 (7) 67.2 (7)	0.3 (0.2) 4.4 (0.8) 0.4 (0.2) 4.3 (0.7)	0.2 (0.4)^[b]^ 18.0 (7.8^[b]^ 25.2 (12)^[b]^ 0.7 (1.5)^[b]^ 20.5 (8.6)^[b]^ 29.1 (12)^[*b*]^	1.6(0.5) 1.8 (0.4) 1.6 (0.5) 1.9 (0.6)	Medicated (OFF) Partly Medicated (OFF)	w/o cognitive impairment Mildly impaired cognition	[FA of YPD] *n.s*. [FA of OPD(Y>3)] genu, body and splenium of CC, CG, SN, midbrain, geniculate nuclei of the thalamus, fronto- parieto- occipito- temporal multifocal non-motor areas	[FA of YPD] CST, ALIC, PLIC, striatum (Put, pallidum, caudate), PTR, SCR, white matter adjacent to SMA, MC and postcentral cortex [FA of OPD(Y0-1)] CST

[a] *Studies using PPMI data. Other studies in the list were using independent data source*.

[b] *Measured by MDS-UPDRS version. UPDRS-III = motor exams (part-III) of the Unified Parkinson's Disease Rating scale (100); MDS-UPDRS-III = motor exams (part-III) of the Movement Disorder Society – sponsored revision of the Unified Parkinson's Disease Rating scale (101). n.s., not significant; WM, white matter; CC, corpus callosum; CST, corticospinal tract; Put, putamen; SN, substantia nigra; MC, motor cortex or precentral gyrus; SMA, supplementary motor areas; IC, internal capsule; EC, external capsule; ALIC, anterior limb of internal capsule; ACR, anterior corona radiata; SS, sagittal stratum; UF, uncinate fasciculus; CG, cingulum, or cingulate tract; FXST, crus of fornix or stria terminalis; SLF, superior longitudinal fasciculus; PTR, posterior thalamic radiation; ILF, inferior longitudinal fasciculus; IFOF, inferior fronto-occipital fasciculus; ATR, anterior thalamic radiation; SCR, superior corona radiata; PLIC, posterior limb of internal capsule; GM, gray matter; SFOF, superior fronto-occipital fasciculus; MCP, middle cerebral peduncle; SCP, superior cerebral peduncle; PD-TD, tremor dominant PD; YPD and OPD, PD onset age ≤ 50 years and > 50 years; YHC and OHC, healthy controls age ≤ 50 years and > 50 years*.

### The Parkinson's Progression Markers Initiative (PPMI) Findings

Previous studies have highlighted a very complex picture of DTI as a PD-specific biomarker. For example, some studies reported that PD exhibits significantly lower FA, but some others showed PD has higher FA than HC subjects. To address if an FA increase or decrease is a characteristic feature of PD, we investigated the temporal changes of FA along the early disease course, by analyzing data from a multicenter, longitudinal PPMI (103) cohort (from now on termed “this study”). The methodological details of data selection, MRI acquisition, image processing, and analyses of this study are described in the Supplementary Material S1–S3. Overall, the PPMI data is characterized as early diagnosed cases (diagnosed within 2 years), drug-naïve (*de-novo*) including 22% patients with onset at young adulthood (i.e., age ≤ 50 years). Figures below depict the findings resulted from this study, as a comparison with the numerous results published previously.

**Figure 1:**
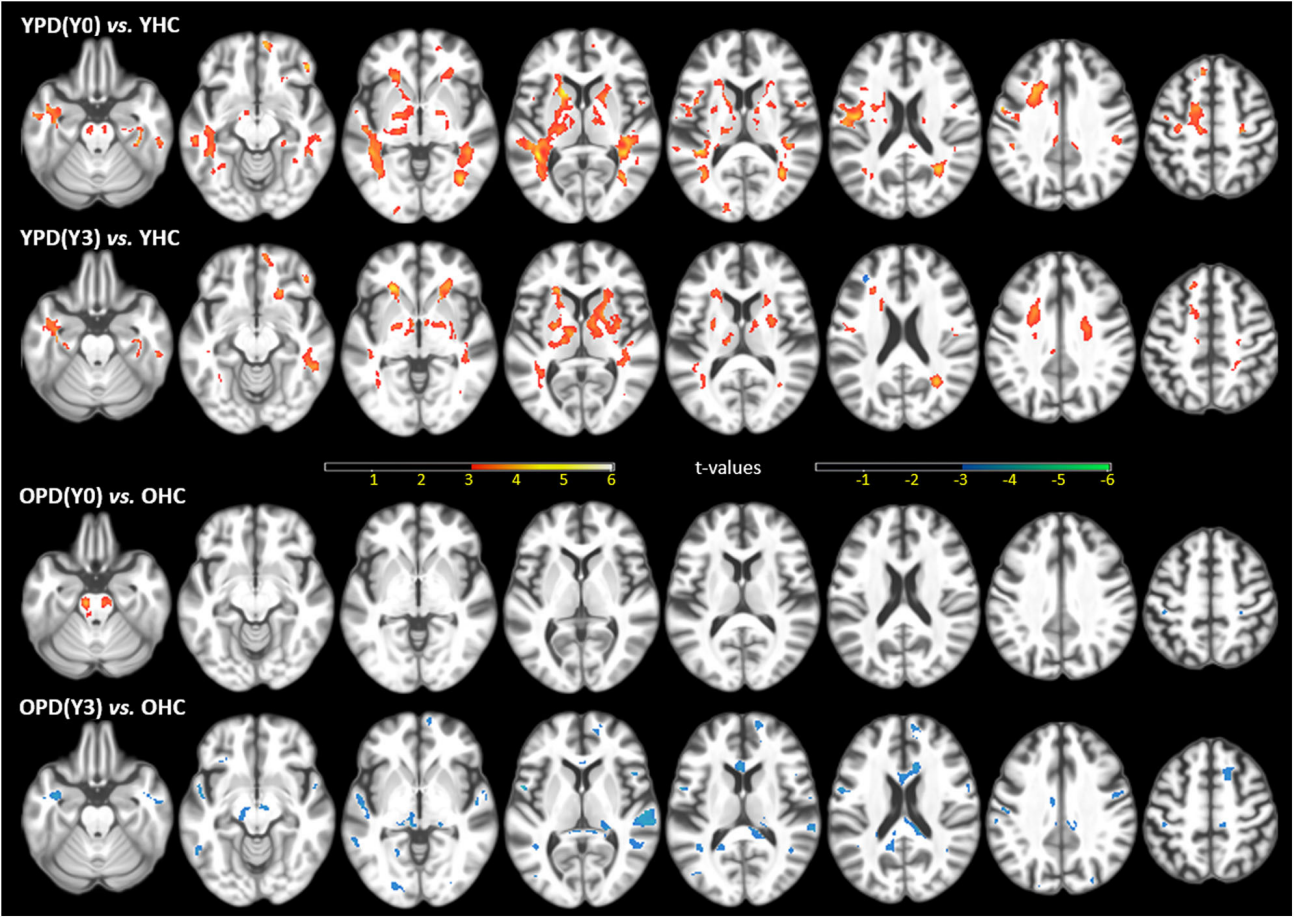
Statistical t-maps of fractional anisotropy (FA) maps, superimposed on a T1WI, of the significant differences between the first-year PD(Y0) or after the 3rd-year PD(Y3) patients and demographically matched HC (ANCOVA *p* ≤ 0.001, uncorrected for multiple comparison), separated for young onset (YPD) and old onset (OPD) groups, and demographically matched young (YHC) and old (OHC) controls. Clusters in warm colors indicate regions of higher FA values in PD than HC; clusters in cool colors indicate the opposite. This figure is illustrative and is created by the author.

Figure 1 shows statistical T-maps of FA differences between PD groups within year-1 (Y0) or more than 3 years (Y3), and demographically matched HC groups. Considering the impact of age at PD onset, the figure illustrated results of group differences separately for young-onset group (YPD, age of onset ≤ 50 years) and typical-onset group (OPD, age of onset > 50 years). In comparison between YPD(Y0) and YHC, significantly higher FA was observed in patients in multiple brain regions, including the bilateral corticospinal tract (CST), internal capsule, striatum (the putamen, pallidum, and caudate nuclei), anterior and posterior and superior corona radiata, as well as in white matter areas that lie next to the motor and supplementary motor cortices. Further, a comparison between YPD(Y3) and YHC showed that higher FA in patients remained significant in the internal capsule, striatum, corona radiata, and motor and supplementary motor white matter areas, but with a lesser extent of distribution. In the comparison between YPD and YHC groups, no region was found with lower-than-normal FA. On the other hand, the comparison between OPD(Y0) and OHC showed significantly higher FA in patients only in the bilateral CST. The comparison between OPD(Y3) and OHC showed prominently lower FA in patients in the SN, midbrain, thalamus, and multifocal non-motor areas of the white matter across all major lobes, while no area was found with higher-than-normal FA.

**Figure 2:**
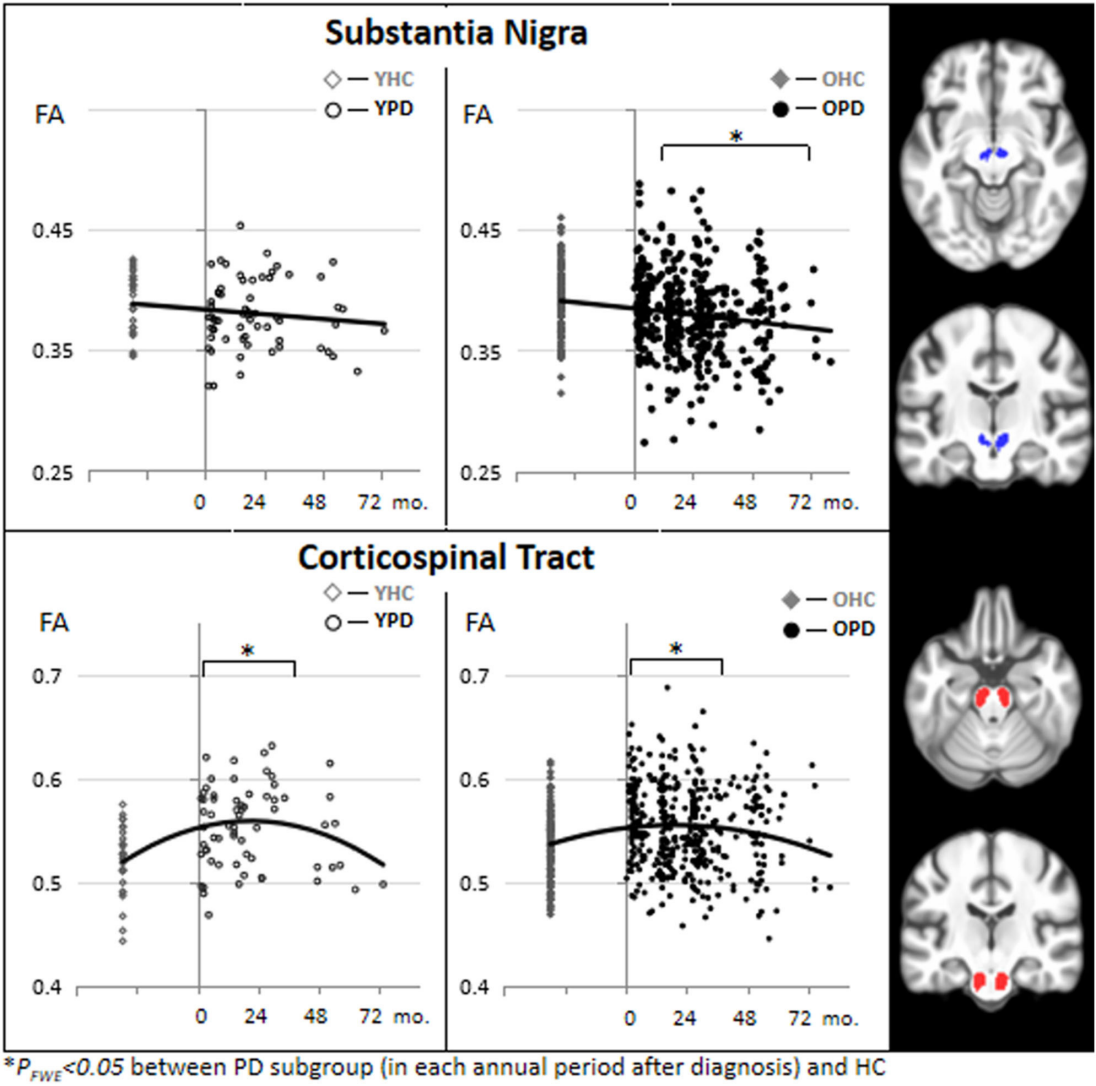
Scatter plots and binominal trend lines of FA changes during the course of PD (from 0 to 72 months after PD diagnosis) in the substantia nigra and the corticospinal tract, separated for young onset (YPD) and old onset (OPD) groups. FA values of the HC subjects are also separated into demographically comparable groups (OHC and YHC). This figure is illustrative and is created by the author.

Figure 2 depicts temporal pattern of FA changes during the early course of PD (from 0 to 79 months after the clinical diagnosis was made) in the SN and CST. For comparison, FA of the HC subjects at baseline are presented. The scatter plots are separately illustrated for the YPD vs. YHC groups and OPD vs. OHC groups. In CST, FA was significantly increased from the first year through the third year for both YPD and OPD, and later decreased to a normal level after the third year of clinical PD. In the SN, FA reduced steadily for OPD throughout the PD course.

Despite a multifocal regional pattern of FA reduction, which has been consistent with those commonly reported in literature, this study presented a robust FA increase in extensive motor areas in a large sample of the earliest and young-onset PD. The FA increase in the CST and other motor areas is not surprising because it has been also reported in previous studies using PPMI data (89, 97, 98) or other pilot data (80, 85, 92). Reduced FA is often found in neurodegenerative conditions, such as aging, Alzheimer's disease, and Parkinsonism, and is believed to be attributed to demyelination, loss of axons, and interrupted connection. It is unlikely that an FA increase is interpreted as its opposite physiological meaning (e.g., axonal regeneration, remyelination, redundant white matter networks) in neurodegenerative disorders. A meta-analysis (104) revealed that previous studies usually considered the FA increase to be due to methodological confounding factors. For example, different MRI scanners, field strength, and the number of diffusion directions could result in variability of the FA measurement and make group comparisons very difficult based on a multi-center population. Another issue is that voxels composed of fiber populations with different spatial orientations (so called “crossing-fibers”) may result in an average increase in FA. There are about 90% white matter voxels containing crossing-fibers (105). Crossing-fibers may affect an anisotropy analysis and might lead to difficulties in interpreting FA increases (106). In addition, a concomitant FA increase may also be due to the variability in ROI sizes, with less or more inclusion of voxels containing surrounding isotropic neurons with low FA. However, the coexistence of FA increase and FA reduction in this study is consistent with the previous meta-analysis (107), which by accounting for heterogeneities of 39 published articles revealed FA increase in the CST and caudate nuclei, and FA decrease in the SN, corpus callosum, cingulate, and temporal areas. With an increasing number of consistent findings, an elevated FA in early PD cannot merely be attributed to methodological confounding factors but could instead indicate some pathophysiological correspondences of the disease. Possible interpretations of the FA increase include compensatory responses or excitatory reactions, which have been proposed by many fMRI (108), perfusion (109), and glucose metabolic (110) studies as a PD-related anatomical pattern of motor dysfunction (PDRD). Compensatory increased structural connectivity could be parallel to hyper-functional activation in similar motor areas (111) and are particularly activated in young aged PD (112). A previous study (98), also using PPMI data, found an FA increase in the tremor dominant (TD) phenotype, but not in the postural instability and gait difficulty (PIGD) phenotype of PD, suggesting that the compensatory white matter reorganization is more specific to the TD type in early PD. Moreover, an earlier study (73) using pilot data reported a whole-brain FA increase only in the akinetic-rigid (AR) phenotype of PD. It is therefore highly encouraged to have more investigations to validate whether the pattern of FA increase is selectively associated with a specific phenotype of motor dysfunction (i.e., TD vs. PIGD or AR subtypes), in addition to several existing pilot cohorts showing conflicting results (18, 19, 41, 113). Although there is a lack of research to date, the pattern of coexisting FA divergency in early PD is likely also explained by the pathophysiology of inhibition (reduced white matter integrity) through direct pathways and disinhibition (increased white matter integrity) through indirect pathways in the basal ganglia (114).

### Hypothesis Driven DTI Analyses in PD

#### DTI Changes in SN and SN Subdivisions

In contrast to the voxel-based whole-brain analysis, the hypothesis-driven region-of-interest (ROI) analysis is often used to identify DTI abnormalities in patients with PD in regions that are known to be specific to the characteristic PD pathologies. SN is one of the main sites of selective loss of dopaminergic neurons in PD, because there is already a loss of 60–80% dopaminergic neurons in the SN before PD motor symptoms emerge (115, 116). Table 4 lists 32 independent studies that analyzed DTI in the total SN area and SN sub-regions in order to identify substantial DTI alterations in patients with PD. These studies were included in four previous meta-analyses and reviews (104, 131, 135, 136). Except for two studies that reported elevated FA values in the SN of PD, 11 studies found no FA abnormalities. The other 19 studies reported significantly reduced FA values in the entire SN. Aside from FA, only a few studies found an MD increase in the SN, while most others found no significant MD abnormalities, suggesting FA is a better contributive index in identifying nigral abnormalities in PD. However, it remains unclear whether or not FA alterations in the entire SN (Type A ROI subdivision as illustrated in Table 4) is sensitive to capture the dopamine loss in PD.

**Table 4:** Overview of studies in comparison of FA/MD differences in the SN and SN subregions between PD and HC.

**First Author, Year**	**No. of PD/HC**	**Age of PD**	**Duration (years)**	**UPDRS-III**	**H&Y**	**ROI sub-division**	**FA decrease**	**MD increase**
Peran et al. (117)	30/22	61.9 (11.1)	4.5 (± 2.5)	12 (± 6)	1.6	Type A1	SN	*n.s*.
Du et al. (118)	16/16		4.8 (3.0)	23.1 (12.3)		Type A1	SN (contra-lateral)	*n.s*.
Zhan et al. (11)	12/20	67.4 (8.0)		26.3 (12.2)		Type A1	SN	*n.s*.
Nagae et al. (113)	21/20	61.1 (7.7)	5.5 (3.4)	31.4 (10)^[b]^	2.2	Type A1	SN (contra-, ipsi-lateral)	SN (ipsi-, contra-lateral)
Johsi et al. (119)	24/26	61.9 (4.9)	2.9 (2.9)	22.2 (11)	1.6	Type A1	*n.s*.	Not tested
Chen et al. (92)	30/30	64.3 (10.3)	5.2 (3.6)	17.9 (9)	1.8	Type A1	*n.s*.	*n.s*.
This study^[a]^	482/146^[a]^	61.5 (9.4)	1.9 (1.4)	23.4 (11)^[b]^	1.7	Type A1	SN	*n.s*.
Yoshikawa et al. (120)	12/8	71.3 (7.7)			1–3	Type A2	SN	Not tested
Chan et al. (121)	73/78	63.6 (9.8)	5.0 (± 4.1)		2.4	Type A2	SN	*n.s*.
Rolheiser et al. (122)	14/14	56.0 (4.8)	2.5 (1.5)		1.3	Type A2	SN	*n.s*.
Wang et al. (123)	30/30	64.5 (3.4)	5.2 (2.0)	33.6 (14.1)	2	Type A2	SN^[c]^	*n.s*.
Skorpil et al. (124)	14/15	64 (43–73)		32.1 (6)	1.75	Type A2	SN	*n.s*.
Perea et al. (125)	12/13	67.5 (4.0)		19.6 (7.1)		Type A2	*n.s*.	*n.s*.
Li et al. (126)	23/23	65.6 (46–77)				Type A2	SN	*n.s*.
Kamagata et al. (61)	58/36	68.8 (7.5)	7.4 (4.4)	18.0 (8.5)	2.5	Type A2	SN (contra-lateral)	SN (contra-, ipsi-, bi-lateral)
Loane et al. (127)	18/14	56.8 (6.8)	3.9 (2.2)	26.2 (9.2)		Type A2	*n.s*.	*n.s*.
Wei et al. (12)	22/22	61.4 (9.7)			<2	Type A2	SN	Not tested
Gattellaro et al. (128)	10/10	63.8 (15.7)				Type A3	not tested	SN
Chan et al. (16)	21/19	72.0 (4.8)			2.2	Type A3	*n.s*.	SN
Jiang et al. (79)	31/34	69.4 (8.0)	4.4	20.4	2.6	Type A3	SN	*n.s*.
Vaillancourt et al. (129)	14/14	57.2 (9.6)	1.3 (± 0.9)	18 (± 8.1)	1.7	Type B1	SN (posterior> middle>anterior)	Not tested
Prakash et al. (8)	11/12	60.4 (9.3)	5.7 (4.2)	23.5 (9.5)	2.1	Type B1	*n.s*.(asymmetric)	*n.s*. (asymmetric)
Schwarz et al. (104)	32/27	64.8 (11.8)	6.4 (± 4.2)	26.1 (13.9)	1.7	Type B1	*n.s*.	SN (all ROIs)
Lenfeldt et al. (130)	122/34	70.3 (9.7)	2.1 (± 2.0)	26.1 (10.6)		Type B1	SN (all ROIs)^[c]^	*n.s*.
Schuff et al. (7)^[a]^	153/67^[a]^	61.0 (10)	<2	22 (9)^[b]^	1.6	Type B1	*n.s*.	*n.s*.
Zhang et al. (53)	72/72	66.8 (5.4)	1.1 (± 0.6)	14.9 (3.9)	1.67	Type B1	SN (averaged ROIs)	Not tested
Hirata et al. (131)	72/42	62.6 (11.8)	5.7 (± 5.5)	28.7 (14)		Type B1	*n.s*.	Not tested
Knossalla et al. (132)	10/10	57.9 (10.6)		12.9 (11)	1.4	Type B1	SNc (posterior)	Not tested
Ofori et al. (55)^[a]^	28/20 78/56^[*a*]^	64.7 (8.2) 61.6 (9.2)	3.4 (± 1.7) 0.7 (± 0.7)	29.8 (9)^[b]^ 22.9 (9)^[*b*]^		Type B2 Type B2	*n.s*. *n.s*.	Not tested Not tested
Du et al. (133)	40/28	60.8 (8.2)	4.2 (± 4.7)	23.5 (15)	1.7	Type C1	SN (inferior)	*n.s*.
Langley et al. (9)	20/17	60.3 (8.4)		23.2 (9)		Type C1	NM SN (inferior)	NM SN (inferior)
Scherfler et al. (36)	16/14	68.1 (6.1)	3.7 (± 3.7)	20 (10.3)	2.3	Type C2	SN (inferior-medial)	SN (middle, inferior-medial)
Menke et al. (134)	10/10	63.7 6.7)	6.1 (± 4.4)		2.3	Type D	*n.s*.	*n.s*.

*ROI subdivision. Type A1: volume-of-interest of whole SN was auto-transformed from standard atlas; Type A2: manually ROI drawn in a middle slice (or a few slices) of SN; Type A3: one ball/cubical/ellipsoid ROI manually I drawn in a middle slice on each side of SN; Type B1: Middle segment of bilateral SN: three (anterior-medial, middle and posterior-lateral), or Type B2: two (anterior-medial and posterior-lateral) ball/cubical ROIs of SN on each side; Type C1: the whole volume of SN was divided into superior (SNr) and inferior (SNc) segments; Type C2: the whole volume of SN was divided into superior, middle and inferior segment; Type D = SN was divided into lateral SNr (lateral part) and SNc (medial part) based on connectivity profiles. n.s., not significant; SN, substantia nigra; NM SN, neuromelanin-portion of SN; SNr, substantia nigra pars reticula; SNc, substantia nigra pars compacta*.

[a] *Studies using PPMI data*.

[b] *Measured by MDS-UPDRS version*.

[c] *PD has significantly increased FA than HC*.

Molecular and neuropathological studies (137) of PD have highlighted that progressive loss of dopaminergic neurons is primarily involved in the pars compacta (SNc), which contains rich pigment neuromelanin formed by dopaminergic neurons, and lies in the inferior and posterior part of the SN, in contrast to the pars reticulata (SNr), which lies lateral to the SNc. Therefore, focusing the microstructural alterations on SN subregions, such as the SNc, would appear to be ideal and vital in identifying dopamine loss-related abnormality. In general practice, it is challenging to delineate the SNc on DTI image because there are no well-defined borders of this substructure. An earlier study (129) reported that reduced FA in the caudal and lateral sub-regions of the SN could completely separate patients with PD from HC with 100% diagnostic accuracy. The authors proposed that FA measurements in the caudal and lateral sub-regions of the SN, presumably coinciding with the histological described SNc location (ROI subdivision Type B1), have the best diagnostic value for PD. However, eight subsequent studies with the same SN subdivision (subdivision Type B) reported discrepant findings: two studies (13, 132) likely replicated the findings of FA reduction in the dorsal lateral sub-region of the SN in PD; five other studies (7, 8, 55, 104, 131) found no significant FA differences between PD and HC in any SN sub-region; and one (130) reported elevated FA in all the SN sub-regions in PD. Technical difficulties outlining the sub-regions may explain these discrepant findings. To combat these issues, several studies (9, 36, 133) have divided the SN into two or three segments from the anterior top position through the posterior bottom position (ROI subdivision Type C). All these studies reported significantly decreased FA in the caudal (or inferior) segment of SN in patients with PD as compared with HC. Among these studies, Langley 2016 (9) used a mask of caudal zone in the SN that was previously established in a brain atlas based on magnetization transfer MRI that is sensitive to neuromelanin. This mask (i.e., the inferior portion of SN) supposedly coincides with the neuromelanin accumulation site. Another study (134) localized SN subfields using DTI tractography to segment the SN into an internal and an external part (ROI subdivision Type D), claiming that each part represents the SNc (the internal SN) and SNr (the external SN), respectively. However, no significant differences in FA or MD values between patients with PD and HC were identified in any of these subdivisions. It is likely that differences in delineating and partitioning the SN sub-regions across DTI studies are largely responsible for the inconsistent findings. Further methodological improvements are needed to ultimately demonstrate that microstructural alterations in SNc are a sensitive marker for diagnosing PD.

#### DTI Changes in Dopaminergic Tracts

**Figure 3:**
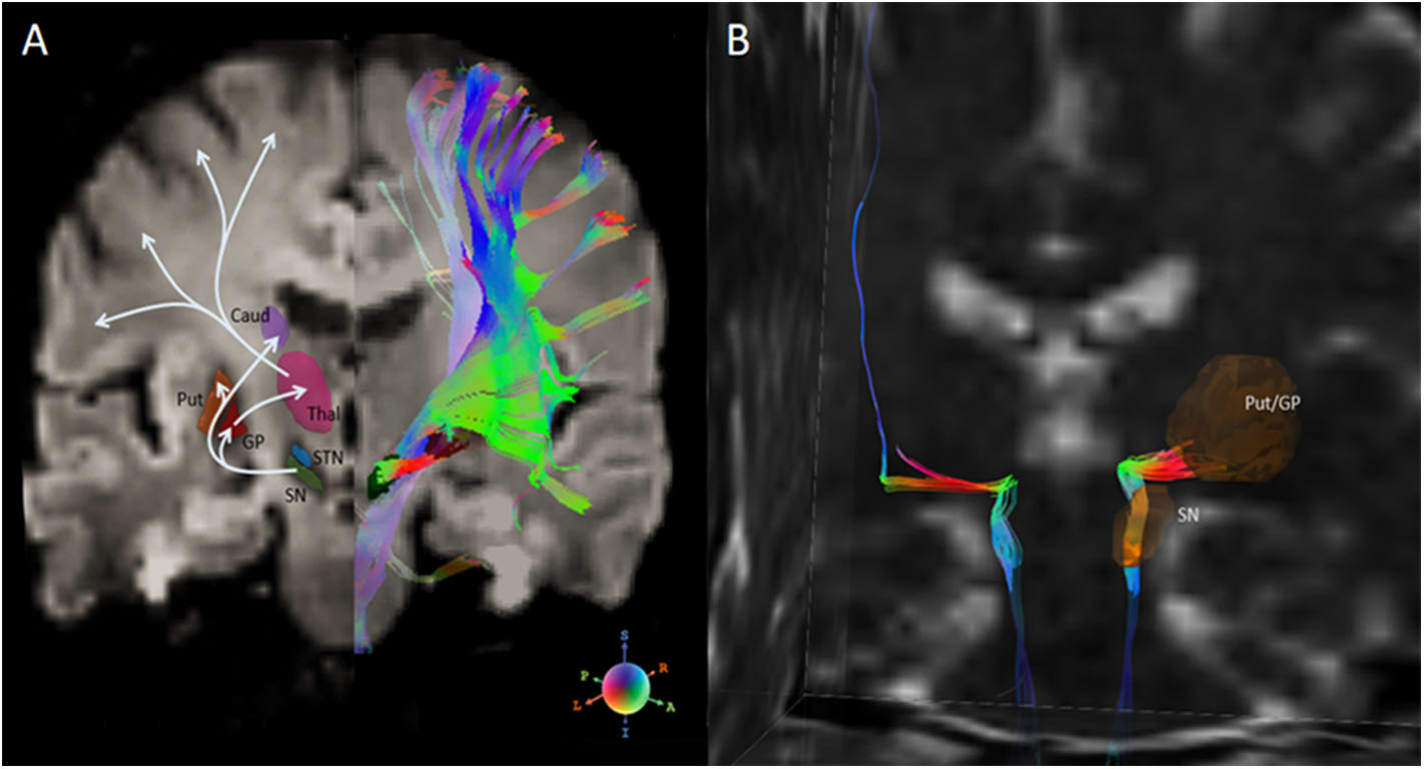
**(A)** Left half-panel: basal ganglia nuclei and connections of the direct and indirect pathways for movement. Right half-panel: diffusion tensor tractography of all fiber streamlines that path through the SN. **(B)** Isolation of the nigrostriatal tract (NST) between SN and inferior Put/GP in a healthy subject. Diffusion tensor tractographic streamlines on the right hemisphere shows the nigrostriatal tract anatomically connects through brainstem, SN and Put/GP. A part of the streamlines further connects through the Thal, and finally projects into the premotor cortex. Fiber orientation is R-G-B coded. Note, DTI tractography does not separate the afferent and efferent directions. SN, substantia nigra; Put, putamen; GP, globus pallidus; Caud, caudate nucleus; Thal, thalamus; STN, subthalamic nuclei. This figure is illustrative and is created by the author.

The classical pathophysiological model of PD and recent updates have been established based on experimental and clinical studies (138–141), which consists of functional disabilities of two main pathways: an “indirect circuit,” which interconnects neocortex-putamen-external pallidum/subthalamic nucleus-SN, and a “direct circuit,” which interconnects neocortex-putamen-internal pallidum-SN-thalamus-neocortex. Under the conditions of PD, the reduced dopamine (results from neuronal loss in the SN) inhibits the indirect pathway, resulting in slow movement, and excites the direct pathway that may lead to unwanted movement such as resting tremor. Figure 3 (left panel) illustrates an example of these basial ganglia pathways on DTI imaging. Neuroanatomically, the dopaminergic pathway, meaning the structural connection between SN and putamen, has been assumed to contain dual effects of activities onto the indirect and direct pathways. Detecting white matter damage to these neuromodulator pathways could add to the understanding of PD pathophysiology.

Diffusion tensor tractography, as an alternative DTI-based approach, offers a possibility to detect the disrupted connections of the dopaminergic pathway and may aid the early diagnosis of PD. White matter tracts that connect the SN and striatum, namely the nigrostriatal tract (NST), are considered one of the major dopaminergic pathways, and have been shown clinically applicable in viewing anatomy of the healthy human brain (142), as well as targeting deep brain stimulation (DBS) (143, 144). Although diffusion tensor tractography could not reach much detailed pathophysiologic meanings due to the limited resolution and directional information, it might still indirectly reflect a functional dysregulation of the dopaminergic circuits on a neuroanatomical basis. Figure 3 (right panel) shows an example of the nigrostriatal tractography in a healthy PPMI subject. An early DTI study (120) measured DTI indices in 11 oval ROIs that were arrayed along a line between the SN and caudate/putamen complex, and reported significantly lower than normal FA values in patients with parkinsonism at all disease stages. With the advances of DTI tractography, recent studies (13, 59, 89, 145, 146) have assessed the FA and tract connectivity profiles of the NST in identifying differences of nigrostriatal connection between patients with PD and healthy subjects. Most of these studies reported either an FA decrease (along with an increase in RD) (13, 59) in PD or reduced tract connectivity (59, 145, 146). Some studies (13, 59, 60) also found that reductions in FA or tract profiles are associated with the severity of motor dysfunctions in PD patients. These consistent findings provide a possible explanation of PD pathophysiological mechanism, that is, the main motor manifestations of PD are related to the diminished connectivity of this dopaminergic circuit. Although based on a small sample, Menke et al. (147) demonstrated that the combination of volumes of the SN and the NST could achieve 100% sensitivity and 80% specificity for PD classification. Taken together, these studies suggest that DTI tractography is a promising, complementary marker of PD diagnosis. On the other hand, a tractographic analysis of the NST also benefits new PD treatment strategies that target the nigrostriatal pathway, such as deep brain stimulation, dopamine graft implantation, and infusion of glial-cell-line–derived neurotrophic factor.

Other white matter circuits that may be involved in PD pathology are of research interests. Braak et al. (3) suggested that Lewy bodies are mainly confined to the medulla oblongata/pontine tegmentum and olfactory bulb/anterior olfactory nucleus in the earliest stages of PD. Using voxel-based analysis, two studies (90, 148) found PD is associated with abnormal diffusivity in white matter tracts adjacent to the olfactory sulcus. Although tractography of the olfactory tract is practically difficult in this area, using ROI drawing, some studies (92, 119, 122) reported low FA of the olfactory tract in PD. Another interesting approach is the tractographic analysis in corticothalamic connections of PD. The reduction of dopamine in SN results in abnormal activation of connections between the thalamus and the motor cortices. This may subsequently lead to inhibition of basal ganglia output and dysfunction in the cortical-subcortical circuits. Several studies have focused on the DTI connection of the corticothalamic tracts (14, 60, 85). However, diverse FA changes were reported in these studies.

## DTI Assessment of Clinical Symptomatology and Dopamine Transporter

PD is characterized as a wide variety of motor and non-motor symptoms, while the neuropathological processes underlying these heterogeneous symptoms are not fully understood. Assessing imaging-clinical correlations in PD has been considered useful for adding further insights into neuropathological underpinnings of PD symptomatology. Table 2 summarizes the findings of correlations between available DTI metrics and clinical assessments.

### DTI Correlates of Motor Dysfunction

A previous article (102) provided a thorough review of the correlations between diffusivity abnormalities and PD motor symptoms, but it lacked information about FA. Many studies, including the analyses with PPMI data, have reported significant correlations between decreased FA in the SN and increased severity of the motor symptoms, which are assessed by the motor exams (part-III) of the Unified Parkinson's Disease Rating Scale (UPDRS) (7–12, 61, 92) or H&Y scales (113, 121). However, some other studies failed to observe significant correlations. One reason for the discrepant reports of previous studies is the variation of “on-medication” or “off-medication” conditions in real-time examination of the PD patients. Dopaminergic treatments have substantial effects on the clinical measurement of motor symptoms such as UPDRS, whereas they have less effects on structural imaging such as DTI. Another complication is that, unlike quantitative imaging measurement, UPDRS is a subject measurement and depends on observers' experiences (149). Nevertheless, the consistent correlation findings from many previous studies imply that nigral FA values could offer an objective assessment of severity of the PD motor dysfunction, when clinical motor measurement is not affected by real-time treatment effects.

Investigations of the correlations between DTI and subtypes of motor dysfunctions remain rudimentary. A few studies revealed significant correlations between DTI changes and severities in terms of freezing of gait (17, 40), risk of falling (16), severity of postural instability and gait disturbance (PIGD) symptoms (18), motor speed and balance (19), and the degree of tremor (41, 42). Some studies, using DTI for classifying PD motor subtypes (73, 95), have found differentiable group effects only, but no substantial clinical correlations were observed in patients. The abnormal DTI changes in specific regions are considered useful biomarkers that characterize PD symptomology or differentiate PD phenotypes, and thus may provide an enormous potential for managing treatment directions of PD. However, it should be cautioned that the diversity of the subtype definition and the substantial instability in motor scoring might hamper the actual imaging-clinical correlations and interpretations. More reliable, objective measures of motor subtypes are necessary to validate these subtype-associated DTI changes conclusively.

### DTI Correlates of Non-motor Dysfunction

Cognitive impairment is one of the major non-motor syndromes in PD. In patients with non-demented PD, the relationships between white matter integrity and global cognition, functions of various cognitive domains have been investigated in multiple studies (as illustrated in Table 2). For example, global cognitive function measured by Mini-Mental State Examination (MMSE) or Montreal Cognitive Assessment (MoCA) has been found to be associated with abnormal DTI variables in the bilateral frontal, parietal, and temporal regions, long association tracts connecting these cortices, and the hippocampus (25, 26, 43, 45, 95). Executive dysfunctions are associated with abnormal DTI variables widely distributed in the parietal and frontal regions (20–23, 43–45, 51). Declined verbal and semantic fluency and visuospatial memory are associated with abnormal diffusion variables in the parietal and frontal regions (23, 24, 44, 51). Worsened performance of language and attention is associated with abnormal diffusivities in frontal and temporal regions and the fornix (46, 47). Memory impairments are correlated with the frontal and hippocampal diffusivity abnormalities (45, 48, 52). Overall, the characteristic white matter abnormalities associated with cognitive impairment in PD can be summarized as follows: multiple regions are involved with a heterogeneous pattern; abnormal diffusion variables are widely distributed in white matter adjacent to cortices and limbic subcortices; the diffusion abnormalities are predominantly shown as the diffusivity changes but not the FA.

DTI correlates with other non-motor manifestations in PD have been comprehensively reviewed in a previous article (102). Briefly, DTI alterations in the thalamus (27), damaged long association tracts connecting to the frontal cortices, are associated with depression (28) and sadness (29). Diffusion changes of the hippocampus are found to be related to the impaired visuospatial memory, leading to visual hallucinations (150) in the advanced stages of PD. Diffusivity changes in the brainstem, midbrain, and pons are associated with autonomic dysfunction during a rapid eye movement (REM) sleep (33), and diffusion alterations of the limbic fornix are associated with excessive daytime sleepiness (32). However, most of these studies have been limited to small sample sizes, resulting in a low statistical power, and conflictions in terms of findings and interpretations. Further replication studies are needed to elucidate these abnormalities in more details and to truly aid the unique insights into PD symptomology.

### DTI Correlates of Striatal DAT-SPECT

Dopamine transporter single photon emission tomography (DAT-SPECT) provides a meaningful measurement of striatal dopaminergic deafferentation. The best quantitative assessment on DAT-SPECT is to measure the striatal dopamine transporters binding ratio (SBR) in the left and right putamen and caudate nucleus. Studies have demonstrated substantial SBR signal loss in early PD patient relative to controls (151, 152). Recent machine learning techniques (such as using the support vector machine, SVM) have shown that the SBR measures achieve high accuracies (97–100%) in predicting early PD (153, 154). Additionally, the SBR has been shown strongly correlated with the number of dopaminergic neurons in substantia nigra (155). Pathological data also confirmed that quantitative measures of SBR enhance the accuracy of detecting dopaminergic neuron loss in PD (156), suggesting that SBR might be a reliable diagnostic biomarker of PD. Therefore, identifying a significant relationship between abnormal DTI and diminished SBR would support the idea that DTI alteration in the dopaminergic area is a potential marker of PD pathology. Recent findings showed a strong relationship between abnormal DTI [including decreased free water (FW) (7, 55) and increased FW (157) of the SN] and decreased putaminal SBR in PD patients. Furthermore, the progressive reduction of nigral FA has been shown correlated with the rate of SBR reduction over the first year of the PPMI patients (158). Aside from a few studies (159, 160) that reported controversial findings, the strong inter-modality correlations suggest that the abnormal DTI changes in the SN are, at least indirectly, associated with the loss of dopaminergic neurons in PD. Thus, this supports the usefulness of nigral DTI measures in linking neurodegeneration to the characterized dopaminergic deficiency in PD.

## DTI Assessment of PD Progression and Treatment Effect

Although it remains a question using the DTI as a diagnostic marker of early PD, many previous studies have reported stable, region-specific, cross-sectional correlations between DTI alterations in the dopaminergic regions and motor symptom severities, suggesting the usefulness of longitudinal DTI as a reliable biomarker for monitoring PD progression. A recent longitudinal DTI study (127) has found increased rate of DTI abnormalities (i.e., FA decrease and MD increase) in the SN of PD patients over a 19.3-month follow-up. Ofori et al. (161) reported significant FW increases in the posterior SN at 1-year follow-up of PD. Further, the same authors' group (162) confirmed these longitudinal FW increases using PPMI data (103 PD at baseline, the 1st, 2nd, and 4th year) in comparison with unchanged FW in the control group (49 HC at baseline, the 1st year). Another longitudinal study (158) measuring FA and diffusivity variables of the PPMI data (122 PD vs. 50 age matched HC at baseline and 1-year follow-up) reported that PD has the highest annual rate of 3.6 ± 1.4% FA reduction in the SN, followed with a moderate rate of FA reduction in the basal ganglia, in comparison with the non-significant DTI changes in HC. Several longitudinal DTI studies identified other vulnerable regions over the PD progression: a recent study (163) found PD had a greater decrease in FA and increase in MD in the rostral brainstem, compared to controls; a 2-years longitudinal DTI study (164) reported a decrease in FA in the putamen of PD patients; another cohort (94) with 18-month follow-up of PD patients with cognitive impairment revealed greater MD increases in frontal white matter than those patients with normal cognition. These studies have presented a similar topology (mainly in the SN, also involved in the midbrain, thalamus, and to some extent to the frontal white matter) of FA decrease/MD increase over the PD course. This anatomical pattern of longitudinal DTI changes is consistent with the regional spread of Lewy body and the accumulation of Lewy neurites during PD progression (5). According to the generally consistent findings, longitudinal DTI shows a promising PD progression marker and could be valuable for monitoring and evaluating treatment effects.

**Figure 4:**
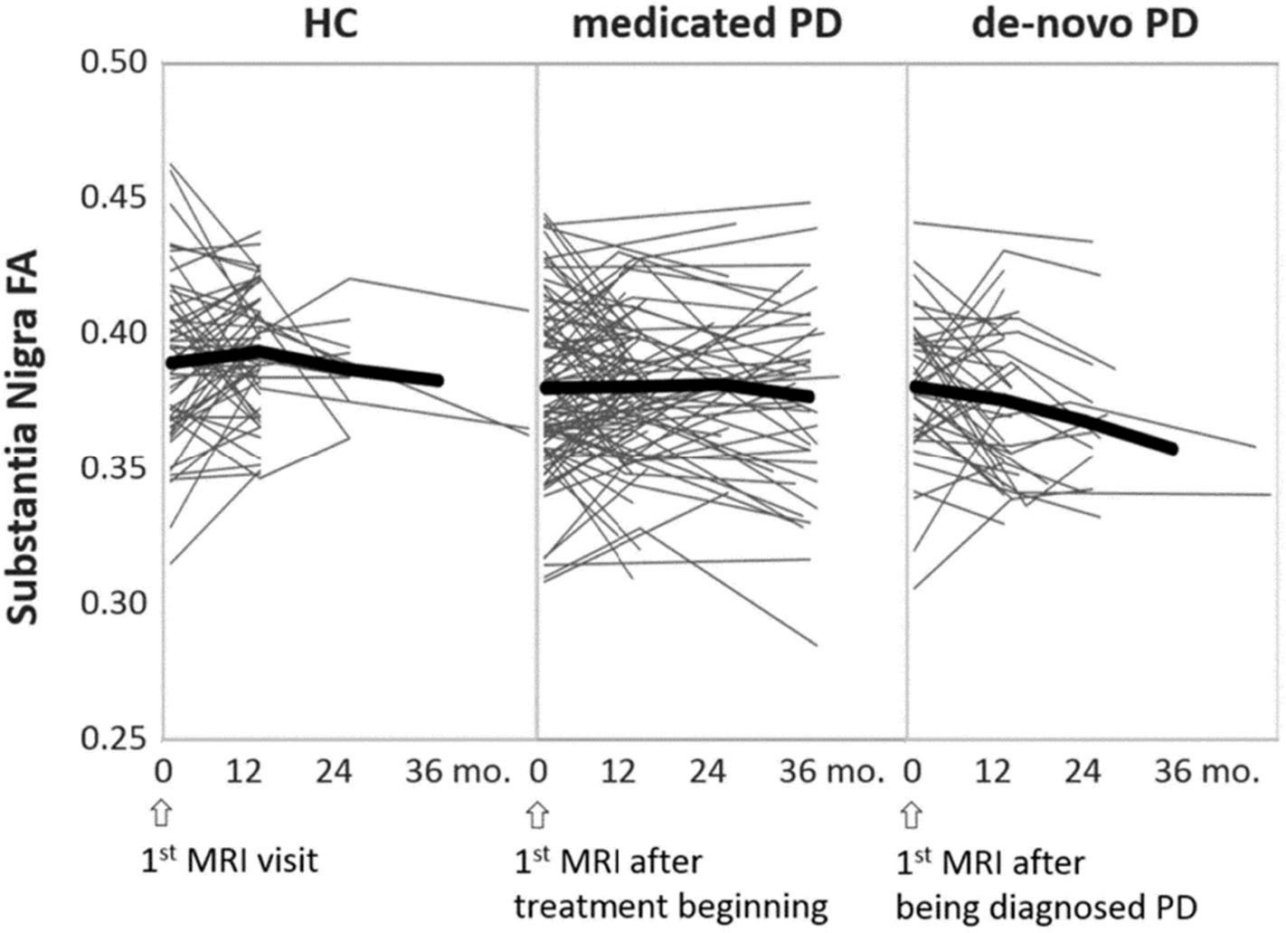
Individual trajectories of the nigral FA changes over time, separately illustrated for HC, treated (medicated), and untreated (*de-novo*) PD groups. The thick black lines indicate the mean trajectory of FA changes over time at each group level. Time is represented as the MRI intervals, starting from the baseline MRI through the latest follow-up MRI. This figure is illustrative and is created by the author.

Outcome measures that are most commonly used for tracking the PD treatment effects are the standard UPDRS scores (165, 166) and, sometimes, the dopamine transporter imaging (167). A PPMI study (168) followed 423 patients from treatment beginning to year-5 and found that dopaminergic therapy provides significant improvements in the Movement Disorder Society revised UPDRS (MDS-UPDRS) scores and the SBR calculated from DAT-SPECT. However, these functional outcome measures may appear significantly different depending on whether they are evaluated at ON or OFF medication status. Tracking the long-term treatment effects using DTI measures could be promising as DTI is used to identify chronic responses of the brain microstructure, and thus is considered to be less affected by the ON or OFF medication status at the imaging time. Figure 4 depicts individual trajectories of nigral FA values extracted from the PPMI data (134 patients and 75 healthy subjects) over a maximum of 36 months (data information is provided in Supplementary Material S1–S3). It shows that the untreated PD patients exhibit on average a steeper decline in FA than HC subjects, while the treated PD patients maintain their respective FA levels to a decline that begins in the 3rd year of medication. This finding supports the fact that early PD is generally responsive to medications. Taken together with recent PPMI findings that nigral FA is associated with disease duration and motor rating scales (97, 169), we suggest that disease modifying effects of levodopa treatment can be measured by longitudinal changes of nigral FA, at least in the first few years of treatment. Further DTI studies of PD clinical trials are needed to validate whether this is indeed true.

## DTI Assessment of Differential Diagnosis

### Differentiation Between PD and Atypical PD

It is important to diagnostically differentiate between idiopathic PD and atypical parkinsonism (aPD), including the three most common sporadic neurodegenerative syndromes: multiple system atrophy (MSA), progressive supranuclear palsy (PSP), and corticobasal syndrome (CBS), because interventional management and prognosis could be different and difficult for aPDs. DTI shows a substantial value in such differentiation.

Compared to idiopathic PD, various DTI studies have demonstrated reduced FA and/or increased MD in the pons, cerebellum, middle cerebellar peduncles (MCP), and putamen in patients with a Parkinsonian type of MSA (MSA-P) (88, 126, 170–174). Several cross-validation studies (173, 175–178) reported that diffusion measurement has 73–94% sensitivity and 89–94% specificity, and 89–100% area under curve (AUC) in discriminating MSA-P from idiopathic PD.

PSP and CBS are neurodegenerative disorders characterized by abnormal tau pathology in the form of globose neurofibrillary tangles, tufted astrocytes, coiled bodies, and threads, with a predominance of 4-repeat (4R) tau isoforms (179). Both diseases provoke symptoms characteristic of PD. A recent review (180) suggests that quantitative measures and visual assessments of atrophy in the superior cerebellar peduncles (SCP) are substantial for differentiating PSP from PD, suggesting microstructural analysis of this key anatomy may provide supportive information. DTI studies (174, 181–183) showed that patients with PSP present strikingly decreased FA and increased MD in the SCP in comparison to patients with idiopathic PD. Studies (174, 184) examining FA of the SCP yielded 86–94% sensitivity and 88–94% specificity for differentiating PSP from PD. Although there are few related investigations in literature, a previous study reported significantly decreased FA and increased MD in the posterior callosal truncus in CBS patients in comparison to PD. Measuring MD of the truncus yielded a 78% AUC in discriminating between CBS and PD.

In summary, PD and aPD are differentiable according to their unique anatomical distributions of microstructural abnormalities detected by reduced FA and increased MD. For example, abnormal DTI in the cerebellum and MCP may be primarily involved in MSA-P; DTI of the SCP is mostly vulnerable in PSP; and abnormal DTI in the supratentorial white matter regions is primarily involved in CBS. Additional evidence that supports these regionally specific changes in PSP and CBS are the high progressive rate of DTI abnormalities (185) and the progressive atrophy (186, 187) in respective key regions of PSP and CBS observed in previous longitudinal imaging studies. For idiopathic PD, the predominant DTI changes are characterized in the SN and the basal ganglia.

### Differentiation Between PD and Associated Cognitive Impairment and Dementia

Cognitive impairment, one of the non-motor symptoms, is particularly problematic in PD. Mild cognitive impairment (MCI) occurs in approximately 30% of PD patients (188). Up to 80% of late-staged PD patients eventually develop an associated dementia (PDD) (189, 190). In general, subtle cognitive deficits occur in early-staged PD patients, and dementia typically occurs in elderly patients or patients in advanced stages. With matched stages and ages, PD patients with MCI (PD-MCI) show a decreased FA and increased MD compared to cognitively normal PD (PD-CN) predominantly in the frontal and interhemispheric white matter (i.e., genu and body of the corpus callosum) (45, 191–193). The PDD group, when compared to the non-demented PD group, showed FA decrease and/or MD increase in the bilateral cingulate tract (194–196), hippocampus (26), prefrontal white matter, and the genu of the corpus callosum (196, 197). In addition, cognitive status, measured by MMSE or MoCA, was associated with abnormal DTI changes in the corpus callosum, anterior cingulate, and several frontal white matter areas (26, 195, 196, 198). Moreover, Shchuz's study (199) suggested that degeneration of the nucleus basalis of Meynert measured by DTI predicts the onset of cognitive impairment, whereas a PPMI study (200) could not confirm DTI as a predictor for further cognitive decline.

The neuropathological basis of dementia in PD is not yet clear. Detectable DTI alterations in the white matter connecting to the neocortex and limbic cortex, including the frontal white matter, the corpus callosum, cingulum, and hippocampus may provide meaningful information in identifying dementia from PD. Consistent with Braak's stages V-VI (3), when α-synuclein spreads to neocortex particularly to the frontal, temporal, and limbic cortices, the abnormal DTI changes in these neuronal fibers play an important role in manifesting dementia processing in PD. On the other hand, the primary mechanisms of cognitive impairment in PD have been manifested by atrophy and reduced glucose hypometabolism in the cerebral cortices (e.g., the temporoparietal cortices) and limbic cortices (e.g., the posterior cingulate gyrus and the hippocampus) (201). White matter burden might occur secondarily or indirectly after the gray matter pathologies such as cortical accumulations of amyloid beta and tau. Further, a study (202) reported that PD with early onset of MCI (onset at PD < 1 year) exhibited lower FA and higher diffusivities in the frontal white matter than PD with late onset of MCI (onset at PD ≥1 year). In this context, whether the cognitive decline and dementia are manifested by age or dopamine deficiency needs further investigations.

A few studies (25, 203) using DTI in differentiation between PDD and Lewy body dementia (LBD) found generally similar regions of reduced FA in both disorders. A few other studies (51, 125) comparing PDD with Alzheimer's disease suggested that PDD may exhibit greater white matter abnormalities than Alzheimer's disease, whereas Novellin et al. (204) suggested the controversial findings. Due to the limited number investigations and the conflicting results, whether DTI can reliably differentiate PDD from LBD or Alzheimer's disease remains unknown.

## Developments in DTI Techniques

### Advanced Diffusion Imaging Technology

Table 5 lists a comparison of imaging parameters in conventional DTI, PPMI, and advanced diffusion imaging techniques. To date, DTI is not broadly applied in clinically standard MRI examinations. In clinical practice, the conventional DTI sequence, especially for scanning patients, has to be performed within 10 min or so. The limited scan time results in problems such as low resolution, noise, artifacts, distortion and crossing-fibers, and reduces qualities of the DTI image and its quantitative measures. Technical developments and limitations of DTI studies in clinical PD have been well-documented in recent review papers (107, 205). New developments of diffusion imaging techniques, such as high angular resolution diffusion imaging (HARDI) (206) and diffusion spectrum imaging (DSI) (207), have shown improvements including increased signal sensitivity and resolution, gaining details of intravoxel directions and allowing a better differentiation of the crossing fibers or the joining fibers. These novel techniques are primarily used for mapping human brain connectome and brain fiber atlas building. Optimization of these sequences to be a clinically tolerable acquisition will be necessary for their clinical application.

**Table 5:** Comparison of DTI scan parameters in conventional DTI, PPMI study, and advanced diffusion imaging techniques.

**Scan parameters**	**Conventional DTI (employed in clinical PD studies)**	**PPMI**	**Sophisticated techniques (e.g., HARDI, DSI)**
Magnetic fields	1.5–3 Tesla	3 Tesla	3,7 Tesla or greater
Number of *b* = 0 (s/mm^2^)	Usually one (can be multiple)	One	One or multiple
Maximum *b* value (s/mm^2^)	800, 1,000, or 2,000	1,000	1,000, 2,000 or more
Spatial resolution	1 × 1 × 2.5–2 × 2 × 2 mm^3^ (or larger)	2 × 2 × 2 mm^3^	3 × 3 × 2 mm^3^ (or larger)
Number of directions	12–80	64	256 and more
Approximate scan time	4–10 min (or longer)	8 min	20–50 min (or longer)
Tractography	Yes (quality depends on direction number)	Yes	Yes, with improved quality
Clinical feasibility	Yes	Yes	Not yet

### Variables Beyond Traditional DTI Indices

Beyond the commonly employed DTI metrics including FA and diffusivities, other quantitative indices that derived from special diffusion sequence or post-image modeling provide additional information on PD biomarker research. FW is an index generated from bi-tensor diffusion model (208) and estimates fractional volume of free water within a voxel without bias from directional dependence and cellular environmental restrictions. FW expresses atrophy-based microstructural degenerations and is also used to correct partial volume effects on FA and MD measures. FW has been found more sensitive than FA in identifying abnormalities in the SN (55), significantly correlated with UPDRS (157), and persistently increased in 1, 2, and 4 years of PD (161, 209). Diffusion kurtosis (210) is an index modeled from multiple *b*-values. Mean kurtosis (MK) estimates non-gaussian forms of diffusion and indicates microstructural complexity such as water leakage through cell membranes and myelin breakdown. It resolves the adverse effects of crossing-fibers, which may influence the accuracy the FA assessment, and thus provide better efficiency in diagnosing early PD (53, 211). More recently, there are increasing interests in the use of structural connectome measures to identify abnormal structural connectivity in PD or in PD motor-subtypes through network-based statistical analysis (57, 63, 212–217). The commonly used indices derived from DTI, together with their biological interpretations and clinical correlations in PD, are summarized in Table 2. Notably, none of the aforementioned variables are specific to PD neuropathology. Postmortem studies are compulsory to confirm these imaging-pathological correlations.

### DTI in Gray Matter

Voxel-based whole brain analyses provide fully automated processing and become popular in DTI studies in PD. However, these analyses have been predominantly focused on white matter structures, because the approaches highly depend on reliable co-registration and spatial normalization methods, while most image registration algorisms used for DTI analyses are established based on white matter skeleton (such as TBSS) or FA intensity, and accompany a use of FA mask (usually sets at absolute FA > 0.2) to constrain DTI analyses within the white matter. These techniques provide substantial improvements of white matter registration, but the accuracy of gray matter registration is sacrificed. Although the motor dysfunctions in PD could be examined through white matter in the pyramidal and extrapyramidal motor pathways, the non-motor symptoms are primarily driven by abnormalities in the cortical and limbic subcortical gray matter (218). Specifically, cortical deficits can be presented in early PD with mild cognitive impairments (219). Therefore, identifying PD-related gray matter microstructural abnormality would be crucial, because it can alert treatment strategies and potentially prevent patients from developing to dementia. Recent technical advantages in DTI processing, including cortical surface-based (220) registration, such as using FreeSurfer (https://surfer.nmr.mgh.harvard.edu/), as well as gray matter skeleton-based registration, such as using Gray matter-based Spatial Statistics (GBSS) (221, 222), permit highly reliable DTI analyses of the gray matter. On the other hand, diffusivity variables are considered having a better sensitivity than FA in capturing neurodegenerative abnormalities in gray matter (223). Using a FreeSurfer registration, and intracortical MD measures of the PPMI data, two recent studies (224, 225) with cross-sectional and longitudinal (within 1-year follow up) analyses have found that compared to controls, PD patients had significantly increased MD in the frontal and occipital cortices; among PD patients these cortical MD changes correlated with worsened cognitive performance; furthermore, the posterior-cortical (i.e., medial temporal and temporo-occipital) MD significantly correlated with the increases of serum neurofilament light chain. These DTI studies in gray matter benefits the understanding of neuropathologic mechanisms underlying PD, especially those accompanied with cognitive impairments.

## Conclusion

This review outlines the clinical utilities of the low-cost, non-invasive diffusion tensor MRI for biomarker of diagnosing PD, correlating PD symptomatology, assessing PD progression, and differentiating atypical types of parkinsonism. The robustness of the findings provides compelling evidences that DTI may be a promising marker for monitoring PD progressing and classifying atypical PD types. Therefore, it provides outcome measures to clinical trials and helps clinicians find better patient management. But the utility of DTI for diagnosing early PD is still challenging. Another merit is that, together with the findings on PPMI data, this review presented a divergent pattern of temporal FA changes in the earliest stage of PD. In particular, FA increase is pronounced in the young onset PD and in the earliest years of PD. These observations help to improve understanding of the pathophysiologic basis (e.g., the compensatory mechanisms, excitations of the inhibitory circuits) during the earliest stages of PD.

Limitations of clinical utilities of DTI shall be mentioned: (1) Because alterations of all DTI variables generally explain the non-specific biological features, they only permit indirect interpretations of the pathogenesis underlying PD. More investigations of imaging-pathological correlations are needed to corroborate a direct implication. Clinical DTI scans are subjected to low resolution, crossing-fibers, noise, and distortions. These factors affect a precise delineation of the key substructures [e.g., SNc, olfactory bulbs and tracts, locus coeruleus, nucleus accumbens, and small fiber connections in the brainstem (226)] that might be specific to PD pathology. Advanced diffusion techniques, such as high-resolution, high filed MRI, improved distortion-corrections, as well as solutions of fiber crossings, will lead to better qualities of DTI analyses, but it is rather important to develop clinically feasible parameters upon these novel techniques. (3) DTI variables are highly dependent on scan parameters, such as MRI field strength, number of encoding directions, and maximum b values. To finally benefit the individual definition and interventions of PD, DTI measures across different MRI centers need to be harmonized, and standardized cutoff points for these DTI measures need to be established.

Further research studies are required to address (1) whether anatomically specific DTI changes are related to the core motor symptom domains (e.g., tremor, rigidity, bradykinesia, gait or postural problems) of PD and how DTI could be able to distinguish these motor subtypes; (2) how DTI change is associated with PD-risks (e.g., prodromal syndromes, risk genes) and whether this DTI change could predict further conversions to PD; (3) whether combining DTI analyses with other imaging modalities (e.g., MRI volumetric and thickness analyses, task-based and resting-state functional MRI, DAT-SPECT and neuroimaging of dopamine or alpha-synuclein, etc.), and clinical evaluations (227) could improve precisions in diagnosing early PD; (4) whether the advanced DTI analyses help the understanding of the brainstem reticular circuits and their roles in the brainstem neurotransmitter systems and how they impact the PD non-motor features (e.g., autonomic syndromes, sleep difficult and sleep behavior problems, pain, fatigue, etc.); and (5) lastly, further validation studies need to take into account of the age of PD onset, disease duration, and whether the non-motor features (especially cognitive deficits) present at the same time as the motor features, whether the PD patients are examined with MRI under an ON or OFF medication status, as well as the history and dosage of medication.

## Author Contributions

YZ: conception and research design, literature review, imaging data processing and analyses, results interpretation, and drafting the first manuscript. MB: conception and research design, results interpretation, language-editing, review, and critical revision of the manuscript. All authors contributed to the article and approved the submitted version.

## Conflict of Interest

The authors declare that the research was conducted in the absence of any commercial or financial relationships that could be construed as a potential conflict of interest.

## References

[1] de Lau LM Breteler MM. Epidemiology of Parkinson's disease. Lancet Neurol. (2006) 5:525–35. 10.1016/S1474-4422(06)70471-9 16713924

[2] DeLong MR. Primate models of movement disorders of basal ganglia origin. Trends Neurosci. (1990) 13:281–5. 10.1016/0166-2236(90)90110-V 1695404

[3] Braak H Ghebremedhin E Rub U Bratzke H Del Tredici K. Stages in the development of Parkinson's disease-related pathology. Cell Tissue Res. (2004) 318:121–34. 10.1007/s00441-004-0956-9 15338272

[4] Saha AR Hill J Utton MA Asuni AA Ackerley S Grierson AJ. Parkinson's disease alpha-synuclein mutations exhibit defective axonal transport in cultured neurons. J Cell Sci. (2004) 117:1017–24. 10.1242/jcs.00967 14996933

[5] Braak H Del Tredici K Rub U de Vos RA Jansen Steur EN Braak E. Staging of brain pathology related to sporadic Parkinson's disease. Neurobiol Aging. (2003) 24:197–211. 10.1016/S0197-4580(02)00065-9 12498954

[6] Moseley ME Kucharczyk J Asgari HS Norman D Anisotropy in diffusion-weighted MRI. Magn Reson Med. (1991) 19:321–6. 10.1002/mrm.1910190222 1652674

[7] Schuff N Wu IW Buckley S Foster ED Coffey CS Gitelman DR. Diffusion imaging of nigral alterations in early Parkinson's disease with dopaminergic deficits. Mov Disord. (2015) 30:1885–92. 10.1002/mds.26325 26260437

[8] Prakash BD Sitoh YY Tan LC Au WL. Asymmetrical diffusion tensor imaging indices of the rostral substantia nigra in Parkinson's disease. Parkinsonism Relat Disord. (2012) 18:1029–33. 10.1016/j.parkreldis.2012.05.021 22705126

[9] Langley J Huddleston DE Merritt M Chen X McMurray R Silver M. Diffusion tensor imaging of the substantia nigra in Parkinson's disease revisited. Hum Brain Mapp. (2016) 37:2547–56. 10.1002/hbm.23192 27029026 PMC4905784

[10] Modrego PJ Fayed N Artal J Olmos S. Correlation of findings in advanced MRI techniques with global severity scales in patients with Parkinson disease. Acad Radiol. (2011) 18:235–41. 10.1016/j.acra.2010.09.022 21232687

[11] Zhan W Kang GA Glass GA Zhang Y Shirley C Millin R. Regional alterations of brain microstructure in Parkinson's disease using diffusion tensor imaging. Mov Disord. (2012) 27:90–7. 10.1002/mds.23917 21850668 PMC4472452

[12] Wei X Yan R Chen Z Weng R Liu X Gao H. Combined diffusion tensor imaging and arterial spin labeling as markers of early parkinson's disease. Sci Rep. (2016) 6:33762. 10.1038/srep33762 27646647 PMC5028727

[13] Zhang Y Wu IW Buckley S Coffey CS Foster E Mendick S. Diffusion tensor imaging of the nigrostriatal fibers in Parkinson's disease. Mov Disord. (2015) 30:1229–36. 10.1002/mds.26251 25920732 PMC4418199

[14] Planetta PJ Schulze ET Geary EK Corcos DM Goldman JG Little DM. Thalamic projection fiber integrity in *de novo* Parkinson disease. AJNR Am J Neuroradiol. (2013) 34:74–9. 10.3174/ajnr.A3178 22766668 PMC3669594

[15] Lenfeldt N Hansson W Larsson A Nyberg L Birgander R Forsgren L. Diffusion tensor imaging and correlations to Parkinson rating scales. J Neurol. (2013) 260:2823–30. 10.1007/s00415-013-7080-2 23974647

[16] Chan LL Ng KM Rumpel H Fook-Chong S Li HH Tan EK. Transcallosal diffusion tensor abnormalities in predominant gait disorder parkinsonism. Parkinsonism Relat Disord. (2014) 20:53–9. 10.1016/j.parkreldis.2013.09.017 24126023

[17] Youn J Lee JM Kwon H Kim JS Son TO Cho JW. Alterations of mean diffusivity of pedunculopontine nucleus pathway in Parkinson's disease patients with freezing of gait. Parkinsonism Relat Disord. (2015) 21:12–7. 10.1016/j.parkreldis.2014.10.003 25455691

[18] Lenfeldt N Holmlund H Larsson A Birgander R Forsgren L. Frontal white matter injuries predestine gait difficulties in Parkinson's disease. Acta Neurol Scand. (2016) 134:210–8. 10.1111/ane.12532 27465659

[19] Surova Y Lampinen B Nilsson M Latt J Hall S Widner H. Alterations of diffusion kurtosis and neurite density measures in deep grey matter and white matter in parkinson's disease. PLoS ONE. (2016) 11:e0157755. 10.1371/journal.pone.0157755 27362763 PMC4928807

[20] Matsui H Nishinaka K Oda M Niikawa H Komatsu K Kubori T. Wisconsin card sorting test in Parkinson's disease: diffusion tensor imaging. Acta Neurol Scand. (2007) 116:108–12. 10.1111/j.1600-0404.2006.00795.x 17661796

[21] Rae CL Correia MM Altena E Hughes LE Barker RA Rowe JB. White matter pathology in Parkinson's disease: the effect of imaging protocol differences and relevance to executive function. Neuroimage. (2012) 62:1675–84. 10.1016/j.neuroimage.2012.06.012 22713671 PMC3413883

[22] Gallagher C Bell B Bendlin B Palotti M Okonkwo O Sodhi A. White matter microstructural integrity and executive function in Parkinson's disease. J Int Neuropsychol Soc. (2013) 19:349–54. 10.1017/S1355617712001373 23321049 PMC3637933

[23] Theilmann RJ Reed JD Song DD Huang MX Lee RR Litvan I. White-matter changes correlate with cognitive functioning in Parkinson's disease. Front Neurol. (2013) 4:37. 10.3389/fneur.2013.00037 23630517 PMC3624087

[24] Chen YS Chen MH Lu CH Chen PC Chen HL Yang IH. Associations among cognitive functions, plasma DNA, and white matter integrity in patients with early-onset Parkinson's disease. Front Neurosci. (2017) 11:9. 10.3389/fnins.2017.00009 28174514 PMC5258716

[25] Hattori T Orimo S Aoki S Ito K Abe O Amano A. Cognitive status correlates with white matter alteration in Parkinson's disease. Hum Brain Mapp. (2012) 33:727–39. 10.1002/hbm.21245 21495116 PMC6870034

[26] Chen B Fan GG Liu H Wang S. Changes in anatomical and functional connectivity of Parkinson's disease patients according to cognitive status. Eur J Radiol. (2015) 84:1318–24. 10.1016/j.ejrad.2015.04.014 25963506

[27] Li W Liu J Skidmore F Liu Y Tian J Li K. White matter microstructure changes in the thalamus in Parkinson disease with depression: a diffusion tensor MR imaging study. AJNR Am J Neuroradiol. (2010) 31:1861–6. 10.3174/ajnr.A2195 20705702 PMC7964038

[28] Huang P Xu X Gu Q Xuan M Yu X Luo W. Disrupted white matter integrity in depressed versus non-depressed Parkinson's disease patients: a tract-based spatial statistics study. J Neurol Sci. (2014) 346:145–8. 10.1016/j.jns.2014.08.011 25194633

[29] Baggio HC Segura B Ibarretxe-Bilbao N Valldeoriola F Marti MJ Compta Y. Structural correlates of facial emotion recognition deficits in Parkinson's disease patients. Neuropsychologia. (2012) 50:2121–8. 10.1016/j.neuropsychologia.2012.05.020 22640663

[30] Chiang PL Chen HL Lu CH Chen PC Chen MH Yang IH. White matter damage and systemic inflammation in Parkinson's disease. BMC Neurosci. (2017) 18:48. 10.1186/s12868-017-0367-y 28595572 PMC5465562

[31] Haghshomar M Rahmani F Hadi Aarabi M Shahjouei S Sobhani S Rahmani M. White matter changes correlates of peripheral neuroinflammation in patients with Parkinson's disease. Neuroscience. (2019) 403:70–8. 10.1016/j.neuroscience.2017.10.050 29126955

[32] Matsui H Nishinaka K Oda M Niikawa H Komatsu K Kubori T. Disruptions of the fornix fiber in Parkinsonian patients with excessive daytime sleepiness. Parkinsonism Relat Disord. (2006) 12:319–22. 10.1016/j.parkreldis.2006.01.007 16621664

[33] Pyatigorskaya N Mongin M Valabregue R Yahia-Cherif L Ewenczyk C Poupon C. Medulla oblongata damage and cardiac autonomic dysfunction in Parkinson disease. Neurology. (2016) 87:2540–5. 10.1212/WNL.0000000000003426 27837003

[34] Ibarretxe-Bilbao N Junque C Marti MJ Valldeoriola F Vendrell P Bargallo N. Olfactory impairment in Parkinson's disease and white matter abnormalities in central olfactory areas: a voxel-based diffusion tensor imaging study. Mov Disord. (2010) 25:1888–94. 10.1002/mds.23208 20669268

[35] Sobhani S Rahmani F Aarabi MH Sadr AV. Exploring white matter microstructure and olfaction dysfunction in early parkinson disease: diffusion MRI reveals new insight. Brain Imaging Behav. (2019) 13:210–9. 10.1007/s11682-017-9781-0 29134611

[36] Scherfler C Esterhammer R Nocker M Mahlknecht P Stockner H Warwitz B. Correlation of dopaminergic terminal dysfunction and microstructural abnormalities of the basal ganglia and the olfactory tract in Parkinson's disease. Brain. (2013) 136:3028–37. 10.1093/brain/awt234 24014521

[37] Wang J Yang QX Sun X Vesek J Mosher Z Vasavada M. MRI evaluation of asymmetry of nigrostriatal damage in the early stage of early-onset Parkinson's disease. Parkinsonism Relat Disord. (2015) 21:590–6. 10.1016/j.parkreldis.2015.03.012 25825242

[38] Esterhammer R Seppi K Reiter E Pinter B Mueller C Kremser C. Potential of diffusion tensor imaging and relaxometry for the detection of specific pathological alterations in Parkinson's Disease (PD). PLoS ONE. (2015) 10:e0145493. 10.1371/journal.pone.0145493 26713760 PMC4705111

[39] Wen MC Heng HS Ng SY Tan LC Chan LL Tan EK. White matter microstructural characteristics in newly diagnosed Parkinson's disease: an unbiased whole-brain study. Sci Rep. (2016) 6:35601. 10.1038/srep35601 27762307 PMC5071859

[40] Iseki K Fukuyama H Oishi N Tomimoto H Otsuka Y Nankaku M. Freezing of gait and white matter changes: a tract-based spatial statistics study. J Clin Mov Disord. (2015) 2:1. 10.1186/s40734-014-0011-2 26788337 PMC4711070

[41] Luo C Song W Chen Q Yang J Gong Q Shang HF. White matter microstructure damage in tremor-dominant Parkinson's disease patients. Neuroradiology. (2017) 59:691–8. 10.1007/s00234-017-1846-7 28540401

[42] Vervoort G Leunissen I Firbank M Heremans E Nackaerts E Vandenberghe W. Structural brain alterations in motor subtypes of Parkinson's disease: evidence from probabilistic tractography and shape analysis. PLoS ONE. (2016) 11:e0157743. 10.1371/journal.pone.0157743 27314952 PMC4912098

[43] Koshimori Y Segura B Christopher L Lobaugh N Duff-Canning S Mizrahi R. Imaging changes associated with cognitive abnormalities in Parkinson's disease. Brain Struct Funct. (2015) 220:2249–61. 10.1007/s00429-014-0785-x 24816399 PMC4485490

[44] Duncan GW Firbank MJ Yarnall AJ Khoo TK Brooks DJ Barker RA. Gray and white matter imaging: a biomarker for cognitive impairment in early Parkinson's disease? Mov Disord. (2016) 31:103–110. 10.1002/mds.26312 26202802

[45] Melzer TR Watts R MacAskill MR Pitcher TL Livingston L Keenan RJ. White matter microstructure deteriorates across cognitive stages in Parkinson disease. Neurology. (2013) 80:1841–9. 10.1212/WNL.0b013e3182929f62 23596076

[46] Zheng Z Shemmassian S Wijekoon C Kim W Bookheimer SY Pouratian N. DTI correlates of distinct cognitive impairments in Parkinson's disease. Hum Brain Mapp. (2014) 35:1325–33. 10.1002/hbm.22256 23417856 PMC3664116

[47] Kantarci K Senjem ML Avula R Zhang B Samikoglu AR Weigand SD. Diffusion tensor imaging and cognitive function in older adults with no dementia. Neurology. (2011) 77:26–34. 10.1212/WNL.0b013e31822313dc 21593440 PMC3127333

[48] Carlesimo GA Piras F Assogna F Pontieri FE Caltagirone C Spalletta G. Hippocampal abnormalities and memory deficits in Parkinson disease: a multimodal imaging study. Neurology. (2012) 78:1939–45. 10.1212/WNL.0b013e318259e1c5 22649213

[49] Haehner A Schopf V Loureiro A Linn J Reichmann H Hummel T. Substantia nigra fractional anisotropy changes confirm the PD at-risk status of patients with idiopathic smell loss. Parkinsonism Relat Disord. (2018) 50:113–6. 10.1016/j.parkreldis.2018.02.026 29477459

[50] Guimaraes RP Campos BM de Rezende TJ Piovesana L Azevedo PC Amato-Filho AC. Is diffusion tensor imaging a good biomarker for early Parkinson's disease? Front Neurol. (2018) 9:626. 10.3389/fneur.2018.00626 30186216 PMC6111994

[51] Auning E Kjaervik VK Selnes P Aarsland D Haram A Bjornerud A. White matter integrity and cognition in Parkinson's disease: a cross-sectional study. BMJ Open. (2014) 4:e003976. 10.1136/bmjopen-2013-003976 24448846 PMC3902504

[52] Gargouri F Gallea C Mongin M Pyatigorskaya N Valabregue R Ewenczyk C. Multimodal magnetic resonance imaging investigation of basal forebrain damage and cognitive deficits in Parkinson's disease. Mov Disord. (2019) 34:516–25. 10.1002/mds.27561 30536444 PMC6590238

[53] Zhang G Zhang Y Zhang C Wang Y Ma G Nie K. Diffusion kurtosis imaging of substantia nigra is a sensitive method for early diagnosis and disease evaluation in Parkinson's disease. Parkinsons Dis. (2015) 2015:207624. 10.1155/2015/207624 26770867 PMC4681830

[54] Kamagata K Zalesky A Hatano T Ueda R Di Biase MA Okuzumi A. Gray matter abnormalities in idiopathic Parkinson's disease: evaluation by diffusional kurtosis imaging and neurite orientation dispersion and density imaging. Hum Brain Mapp. (2017) 38:3704–22. 10.1002/hbm.23628 28470878 PMC6867088

[55] Ofori E Pasternak O Planetta PJ Burciu R Snyder A Febo M. Increased free water in the substantia nigra of Parkinson's disease: a single-site and multi-site study. Neurobiol Aging. (2015) 36:1097–104. 10.1016/j.neurobiolaging.2014.10.029 25467638 PMC4315708

[56] Planetta PJ Ofori E Pasternak O Burciu RG Shukla P DeSimone JC. Free-water imaging in Parkinson's disease and atypical parkinsonism. Brain. (2016) 139:495–508. 10.1093/brain/awv361 26705348 PMC5790142

[57] Kim M Park H. Structural connectivity profile of scans without evidence of dopaminergic deficit (SWEDD) patients compared to normal controls and Parkinson's disease patients. Springerplus. (2016) 5:1421. 10.1186/s40064-016-3110-8 27625975 PMC5001967

[58] Wen MC Xu Z Lu Z Chan LL Tan EK Tan LCS. Microstructural network alterations of olfactory dysfunction in newly diagnosed Parkinson's disease. Sci Rep. (2017) 7:12559. 10.1038/s41598-017-12947-7 28970540 PMC5624890

[59] Tan WQ Yeoh CS Rumpel H Nadkarni N Lye WK Tan EK. Deterministic tractography of the nigrostriatal-nigropallidal pathway in parkinson's disease. Sci Rep. (2015) 5:17283. 10.1038/srep17283 26619969 PMC4664862

[60] Son SJ Kim M Park H. Imaging analysis of Parkinson's disease patients using SPECT and tractography. Sci Rep. (2016) 6:38070. 10.1038/srep38070 27901100 PMC5128922

[61] Kamagata K Hatano T Okuzumi A Motoi Y Abe O Shimoji K. Neurite orientation dispersion and density imaging in the substantia nigra in idiopathic Parkinson disease. Eur Radiol. (2016) 26:2567–77. 10.1007/s00330-015-4066-8 26515546

[62] Kamagata K Zalesky A Hatano T Di Biase MA El Samad O Saiki S. Connectome analysis with diffusion MRI in idiopathic Parkinson's disease: evaluation using multi-shell, multi-tissue, constrained spherical deconvolution. Neuroimage Clin. (2018) 17:518–29. 10.1016/j.nicl.2017.11.007 29201640 PMC5700829

[63] Abbasi N Mohajer B Abbasi S Hasanabadi P Abdolalizadeh A Rajimehr R. Relationship between cerebrospinal fluid biomarkers and structural brain network properties in Parkinson's disease. Mov Disord. (2018) 33:431–9. 10.1002/mds.27284 29436735

[64] Basser PJ Jones DK. Diffusion-tensor MRI. Theory, experimental design and data analysis - a technical review. NMR Biomed. (2002) 15:456–67. 10.1002/nbm.783 12489095

[65] Pierpaoli C Jezzard P Basser PJ Barnett A Di Chiro G. Diffusion tensor MR imaging of the human brain. Radiology. (1996) 201:637–48. 10.1148/radiology.201.3.8939209 8939209

[66] Song SK Sun SW Ramsbottom MJ Chang C Russell J Cross AH. Dysmyelination revealed through MRI as increased radial (but unchanged axial) diffusion of water. Neuroimage. (2002) 17:1429–36. 10.1006/nimg.2002.1267 12414282

[67] Kim JH Budde MD Liang HF Klein RS Russell JH Cross AH. Detecting axon damage in spinal cord from a mouse model of multiple sclerosis. Neurobiol Dis. (2006) 21:626–32. 10.1016/j.nbd.2005.09.009 16298135

[68] Wheeler-Kingshott CA Cercignani M About “axial” and “radial” diffusivities. Magn Reson Med. (2009) 61:1255–60. 10.1002/mrm.21965 19253405

[69] Acosta-Cabronero J Williams GB Pengas G Nestor PJ. Absolute diffusivities define the landscape of white matter degeneration in Alzheimer's disease. Brain. (2010) 133:529–39. 10.1093/brain/awp257 19914928

[70] Thomalla G Glauche V Koch MA Beaulieu C Weiller C Rother J. Diffusion tensor imaging detects early Wallerian degeneration of the pyramidal tract after ischemic stroke. Neuroimage. (2004) 22:1767–74. 10.1016/j.neuroimage.2004.03.041 15275932

[71] Boska MD Hasan KM Kibuule D Banerjee R McIntyre E Nelson JA. Quantitative diffusion tensor imaging detects dopaminergic neuronal degeneration in a murine model of Parkinson's disease. Neurobiol Dis. (2007) 26:590–6. 10.1016/j.nbd.2007.02.010 17428671 PMC2040046

[72] Karagulle Kendi AT Lehericy S Luciana M Ugurbil K Tuite P. Altered diffusion in the frontal lobe in Parkinson disease. AJNR Am J Neuroradiol. (2008) 29:501–5. 10.3174/ajnr.A0850 18202242 PMC8118887

[73] Tessa C Giannelli M Della Nave R Lucetti C Berti C Ginestroni A. A whole-brain analysis in *de novo* Parkinson disease. AJNR Am J Neuroradiol. (2008) 29:674–80. 10.3174/ajnr.A0900 18184843 PMC7978177

[74] Soares JM Marques P Alves V Sousa N. A hitchhiker's guide to diffusion tensor imaging. Front Neurosci. (2013) 7:31. 10.3389/fnins.2013.00031 23486659 PMC3594764

[75] Smith SM Jenkinson M Johansen-Berg H Rueckert D Nichols TE Mackay CE. Tract-based spatial statistics: voxelwise analysis of multi-subject diffusion data. Neuroimage. (2006) 31:1487–505. 10.1016/j.neuroimage.2006.02.024 16624579

[76] Zhang K Yu C Zhang Y Wu X Zhu C Chan P. Voxel-based analysis of diffusion tensor indices in the brain in patients with Parkinson's disease. Eur J Radiol. (2011) 77:269–73. 10.1016/j.ejrad.2009.07.032 19692193

[77] Kim HJ Kim SJ Kim HS Choi CG Kim N Han S. Alterations of mean diffusivity in brain white matter and deep gray matter in Parkinson's disease. Neurosci Lett. (2013) 550:64–8. 10.1016/j.neulet.2013.06.050 23831353

[78] Diez-Cirarda M Ojeda N Pena J Cabrera-Zubizarreta A Gomez-Beldarrain MA Gomez-Esteban JC. Neuroanatomical correlates of theory of mind deficit in Parkinson's disease: a multimodal imaging study. PLoS ONE. (2015) 10:e0142234. 10.1371/journal.pone.0142234 26559669 PMC4641650

[79] Jiang MF Shi F Niu GM Xie SH Yu SY. A novel method for evaluating brain function and microstructural changes in Parkinson's disease. Neural Regen Res. (2015) 10:2025–2032. 10.4103/1673-5374.172322 26889194 PMC4730830

[80] Skidmore FM Spetsieris PG Anthony T Cutter GR von Deneen KM Liu Y. A full-brain, bootstrapped analysis of diffusion tensor imaging robustly differentiates Parkinson disease from healthy controls. Neuroinformatics. (2015) 13:7–18. 10.1007/s12021-014-9222-9 24974315 PMC4498392

[81] Vercruysse S Leunissen I Vervoort G Vandenberghe W Swinnen S Nieuwboer A. Microstructural changes in white matter associated with freezing of gait in Parkinson's disease. Mov Disord. (2015) 30:567–76. 10.1002/mds.26130 25640958

[82] Yoo HB Lee JY Lee JS Kang H Kim YK Song IC. Whole-brain diffusion-tensor changes in parkinsonian patients with impulse control disorders. J Clin Neurol. (2015) 11:42–7. 10.3988/jcn.2015.11.1.42 25628736 PMC4302178

[83] Price CC Tanner J Nguyen PT Schwab NA Mitchell S Slonena E. Gray and white matter contributions to cognitive frontostriatal deficits in non-demented Parkinson's disease. PLoS ONE. (2016) 11:e0147332. 10.1371/journal.pone.0147332 26784744 PMC4718544

[84] Lim JS Shin SA Lee JY Nam H Lee JY Kim YK. Neural substrates of rapid eye movement sleep behavior disorder in Parkinson's disease. Parkinsonism Relat Disord. (2016) 23:31–36. 10.1016/j.parkreldis.2015.11.027 26678512

[85] Mole JP Subramanian L Bracht T Morris H Metzler-Baddeley C Linden DE Increased fractional anisotropy in the motor tracts of Parkinson's disease suggests compensatory neuroplasticity or selective neurodegeneration. Eur Radiol. (2016) 26:3327–35. 10.1007/s00330-015-4178-1 26780637 PMC5021738

[86] Wang M Jiang S Yuan Y Zhang L Ding J Wang J. Alterations of functional and structural connectivity of freezing of gait in Parkinson's disease. J Neurol. (2016) 263:1583–92. 10.1007/s00415-016-8174-4 27230857

[87] Canu E Agosta F Markovic V Petrovic I Stankovic I Imperiale F. White matter tract alterations in Parkinson's disease patients with punding. Parkinsonism Relat Disord. (2017) 43:85–91. 10.1016/j.parkreldis.2017.07.025 28780181

[88] Chen B Fan G Sun W Shang X Shi S Wang S. Usefulness of diffusion-tensor MRI in the diagnosis of Parkinson variant of multiple system atrophy and Parkinson's disease: a valuable tool to differentiate between them? Clin Radiol. (2017) 72:610 e619–e15. 10.1016/j.crad.2017.02.005 28318507

[89] Cousineau M Jodoin PM Morency FC Rozanski V Grand'Maison M Bedell BJ. A test-retest study on Parkinson's PPMI dataset yields statistically significant white matter fascicles. Neuroimage Clin. (2017) 16:222–33. 10.1016/j.nicl.2017.07.020 28794981 PMC5547250

[90] Georgiopoulos C Warntjes M Dizdar N Zachrisson H Engstrom M Haller S. Olfactory impairment in parkinson's disease studied with diffusion tensor and magnetization transfer imaging. J Parkinsons Dis. (2017) 7:301–11. 10.3233/JPD-161060 28482644 PMC5438470

[91] Lee WW Yoon EJ Lee JY Park SW Kim YK. Visual hallucination and pattern of brain degeneration in Parkinson's disease. Neurodegener Dis. (2017) 17:63–72. 10.1159/000448517 27760431

[92] Chen NK Chou YH Sundman M Hickey P Kasoff WS Bernstein A. Alteration of diffusion-tensor magnetic resonance imaging measures in brain regions involved in early stages of Parkinson's disease. Brain Connect. (2018) 8:343–9. 10.1089/brain.2017.0558 29877094 PMC6103245

[93] Li XR Ren YD Cao B Huang XL. Analysis of white matter characteristics with tract-based spatial statistics according to diffusion tensor imaging in early Parkinson's disease. Neurosci Lett. (2018) 675:127–32. 10.1016/j.neulet.2017.11.064 29199095

[94] Minett T Su L Mak E Williams G Firbank M Lawson RA. Longitudinal diffusion tensor imaging changes in early Parkinson's disease: ICICLE-PD study. J Neurol. (2018) 265:1528–39. 10.1007/s00415-018-8873-0 29696499

[95] Pietracupa S Suppa A Upadhyay N Gianni C Grillea G Leodori G. Freezing of gait in Parkinson's disease: gray and white matter abnormalities. J Neurol. (2018) 265:52–62. 10.1007/s00415-017-8654-1 29128929

[96] Rektor I Svatkova A Vojtisek L Zikmundova I Vanicek J Kiraly A. White matter alterations in Parkinson's disease with normal cognition precede grey matter atrophy. PLoS ONE. (2018) 13:e0187939. 10.1371/journal.pone.0187939 29304183 PMC5755732

[97] Taylor KI Sambataro F Boess F Bertolino A Dukart J. Progressive decline in gray and white matter integrity in *de novo* parkinson's disease: an analysis of longitudinal parkinson progression markers initiative diffusion tensor imaging data. Front Aging Neurosci. (2018) 10:318. 10.3389/fnagi.2018.00318 30349475 PMC6186956

[98] Wen MC Heng HSE Lu Z Xu Z Chan LL Tan EK. Differential white matter regional alterations in motor subtypes of early drug-naive parkinson's disease patients. Neurorehabil Neural Repair. (2018) 32:129–41. 10.1177/1545968317753075 29347868

[99] Guan X Huang P Zeng Q Liu C Wei H Xuan M. Quantitative susceptibility mapping as a biomarker for evaluating white matter alterations in Parkinson's disease. Brain Imaging Behav. (2019) 13:220–31. 10.1007/s11682-018-9842-z 29417492

[100] Movement Disorder Society Task Force on Rating Scales for Parkinson's D. The Unified Parkinson's Disease Rating Scale (UPDRS): status and recommendations. Mov Disord. (2003) 18:738–50. 10.1002/mds.10473 12815652

[101] Goetz CG Fahn S Martinez-Martin P Poewe W Sampaio C Stebbins GT. Movement Disorder Society-sponsored revision of the Unified Parkinson's Disease Rating Scale (MDS-UPDRS): Process, format, and clinimetric testing plan. Mov Disord. (2007) 22:41–7. 10.1002/mds.21198 17115387

[102] Hall JM Ehgoetz Martens KA Walton CC O'Callaghan C Keller PE Lewis SJ. Diffusion alterations associated with Parkinson's disease symptomatology: a review of the literature. Parkinsonism Relat Disord. (2016) 33:12–26. 10.1016/j.parkreldis.2016.09.026 27765426

[103] Marek K The Parkinson Progression Marker Initiative (PPMI). Prog Neurobiol. (2011) 95:629–35. 10.1016/j.pneurobio.2011.09.005 21930184 PMC9014725

[104] Schwarz ST Abaei M Gontu V Morgan PS Bajaj N Auer DP. Diffusion tensor imaging of nigral degeneration in Parkinson's disease: a region-of-interest and voxel-based study at 3 T and systematic review with meta-analysis. Neuroimage Clin. (2013) 3:481–8. 10.1016/j.nicl.2013.10.006 24273730 PMC3830065

[105] Jeurissen B Leemans A Tournier JD Jones DK Sijbers J. Investigating the prevalence of complex fiber configurations in white matter tissue with diffusion magnetic resonance imaging. Hum Brain Mapp. (2013) 34:2747–66. 10.1002/hbm.22099 22611035 PMC6870534

[106] Mori S Introduction to Diffusion Tensor Imaging. 1st Edn. Elsevier Science (2007). 10.1016/b978-0-444-52828-5.x5014-5

[107] Atkinson-Clement C Pinto S Eusebio A Coulon O. Diffusion tensor imaging in Parkinson's disease: review and meta-analysis. Neuroimage Clin. (2017) 16:98–110. 10.1016/j.nicl.2017.07.011 28765809 PMC5527156

[108] Baudrexel S Witte T Seifried C von Wegner F Beissner F Klein JC. Resting state fMRI reveals increased subthalamic nucleus-motor cortex connectivity in Parkinson's disease. Neuroimage. (2011) 55:1728–38. 10.1016/j.neuroimage.2011.01.017 21255661

[109] Melzer TR Watts R MacAskill MR Pearson JF Rueger S Pitcher TL. Arterial spin labelling reveals an abnormal cerebral perfusion pattern in Parkinson's disease. Brain. (2011) 134:845–55. 10.1093/brain/awq377 21310726 PMC3105489

[110] Ma Y Tang C Spetsieris PG Dhawan V Eidelberg D. Abnormal metabolic network activity in Parkinson's disease: test-retest reproducibility. J Cereb Blood Flow Metab. (2007) 27:597–605. 10.1038/sj.jcbfm.9600358 16804550 PMC4455600

[111] Collier TJ Lipton J Daley BF Palfi S Chu Y Sortwell C. Aging-related changes in the nigrostriatal dopamine system and the response to MPTP in nonhuman primates: diminished compensatory mechanisms as a prelude to parkinsonism. Neurobiol Dis. (2007) 26:56–65. 10.1016/j.nbd.2006.11.013 17254792 PMC1899875

[112] Hindle JV. Ageing, neurodegeneration and Parkinson's disease. Age Ageing. (2010) 39:156–61. 10.1093/ageing/afp223 20051606

[113] Nagae LM Honce JM Tanabe J Shelton E Sillau SH Berman BD. Microstructural changes within the basal ganglia differ between Parkinson disease subtypes. Front Neuroanat. (2016) 10:17. 10.3389/fnana.2016.00017 26941615 PMC4763054

[114] Alexander GE. Biology of Parkinson's disease: pathogenesis and pathophysiology of a multisystem neurodegenerative disorder. Dialogues Clin Neurosci. (2004) 6:259–80. 22033559 10.31887/DCNS.2004.6.3/galexander PMC3181806

[115] Marsden CD. Parkinson's disease. Lancet. (1990) 335:948–52. 10.1016/0140-6736(90)91006-V 1691427

[116] Cheng HC Ulane CM Burke RE. Clinical progression in Parkinson disease and the neurobiology of axons. Ann Neurol. (2010) 67:715–25. 10.1002/ana.21995 20517933 PMC2918373

[117] Peran P Cherubini A Assogna F Piras F Quattrocchi C Peppe A. Magnetic resonance imaging markers of Parkinson's disease nigrostriatal signature. Brain. (2010) 133:3423–33. 10.1093/brain/awq212 20736190

[118] Du G Lewis MM Styner M Shaffer ML Sen S Yang QX. Combined R2^*^ and diffusion tensor imaging changes in the substantia nigra in Parkinson's disease. Mov Disord. (2011) 26:1627–32. 10.1002/mds.23643 21618607 PMC3154471

[119] Joshi N Rolheiser TM Fisk JD McKelvey JR Schoffer K Phillips G Lateralized microstructural changes in early-stage Parkinson's disease in anterior olfactory structures, but not in substantia nigra. J Neurol. (2017) 264:1497–505. 10.1007/s00415-017-8555-3 28653210

[120] Yoshikawa K Nakata Y Yamada K Nakagawa M. Early pathological changes in the parkinsonian brain demonstrated by diffusion tensor MRI. J Neurol Neurosurg Psychiatr. (2004) 75:481–4. 10.1136/jnnp.2003.021873 14966170 PMC1738942

[121] Chan LL Rumpel H Yap K Lee E Loo HV Ho GL. Case control study of diffusion tensor imaging in Parkinson's disease. J Neurol Neurosurg Psychiatr. (2007) 78:1383–6. 10.1136/jnnp.2007.121525 17615165 PMC2095589

[122] Rolheiser TM Fulton HG Good KP Fisk JD McKelvey JR Scherfler C. Diffusion tensor imaging and olfactory identification testing in early-stage Parkinson's disease. J Neurol. (2011) 258:1254–60. 10.1007/s00415-011-5915-2 21287185

[123] Wang JJ Lin WY Lu CS Weng YH Ng SH Wang CH. Parkinson disease: diagnostic utility of diffusion kurtosis imaging. Radiology. (2011) 261:210–7. 10.1148/radiol.11102277 21771952

[124] Skorpil M Soderlund V Sundin A Svenningsson P. MRI diffusion in Parkinson's disease: using the technique's inherent directional information to study the olfactory bulb and substantia nigra. J Parkinsons Dis. (2012) 2:171–80. 10.3233/JPD-2012-12091 23939442

[125] Perea RD Rada RC Wilson J Vidoni ED Morris JK Lyons KE. A comparative white matter study with Parkinson's disease, Parkinson's disease with dementia and Alzheimer's disease. J Alzheimers Dis Parkinsonism. (2013) 3:123. 10.4172/2161-0460.1000123 24724042 PMC3979316

[126] Ji L Wang Y Zhu D Liu W Shi J. White matter differences between multiple system atrophy (parkinsonian type) and Parkinson's disease: a diffusion tensor image study. Neuroscience. (2015) 305:109–16. 10.1016/j.neuroscience.2015.07.060 26215920

[127] Loane C Politis M Kefalopoulou Z Valle-Guzman N Paul G Widner H. Aberrant nigral diffusion in Parkinson's disease: a longitudinal diffusion tensor imaging study. Mov Disord. (2016) 31:1020–6. 10.1002/mds.26606 27104232

[128] Gattellaro G Minati L Grisoli M Mariani C Carella F Osio M. White matter involvement in idiopathic Parkinson disease: a diffusion tensor imaging study. AJNR Am J Neuroradiol. (2009) 30:1222–6. 10.3174/ajnr.A1556 19342541 PMC7051338

[129] Vaillancourt DE Spraker MB Prodoehl J Abraham I Corcos DM Zhou XJ. High-resolution diffusion tensor imaging in the substantia nigra of *de novo* Parkinson disease. Neurology. (2009) 72:1378–84. 10.1212/01.wnl.0000340982.01727.6e 19129507 PMC2677508

[130] Lenfeldt N Larsson A Nyberg L Birgander R Forsgren L. Fractional anisotropy in the substantia nigra in Parkinson's disease: a complex picture. Eur J Neurol. (2015) 22:1408–14. 10.1111/ene.12760 26118635

[131] Hirata FCC Sato JR Vieira G Lucato LT Leite CC Bor-Seng-Shu E. Substantia nigra fractional anisotropy is not a diagnostic biomarker of Parkinson's disease: a diagnostic performance study and meta-analysis. Eur Radiol. (2017) 27:2640–8. 10.1007/s00330-016-4611-0 27709279

[132] Knossalla F Kohl Z Winkler J Schwab S Schenk T Engelhorn T. High-resolution diffusion tensor-imaging indicates asymmetric microstructural disorganization within substantia nigra in early Parkinson's disease. J Clin Neurosci. (2018) 50:199–202. 10.1016/j.jocn.2018.01.023 29366621

[133] Du G Lewis MM Sen S Wang J Shaffer ML Styner M. Imaging nigral pathology and clinical progression in Parkinson's disease. Mov Disord. (2012) 27:1636–43. 10.1002/mds.25182 23008179 PMC3510346

[134] Menke RA Jbabdi S Miller KL Matthews PM Zarei M. Connectivity-based segmentation of the substantia nigra in human and its implications in Parkinson's disease. Neuroimage. (2010) 52:1175–80. 10.1016/j.neuroimage.2010.05.086 20677376

[135] Deng XY Wang L Yang TT Li R Yu G. A meta-analysis of diffusion tensor imaging of substantia nigra in patients with Parkinson's disease. Sci Rep. (2018) 8:2941. 10.1038/s41598-018-20076-y 29440768 PMC5811437

[136] Cochrane CJ Ebmeier KP. Diffusion tensor imaging in parkinsonian syndromes: a systematic review and meta-analysis. Neurology. (2013) 80:857–64. 10.1212/WNL.0b013e318284070c 23439701 PMC3598454

[137] Fearnley JM Lees AJ. Ageing and Parkinson's disease: substantia nigra regional selectivity. Brain. (1991) 114:2283–301. 10.1093/brain/114.5.2283 1933245

[138] Rodriguez-Oroz MC Jahanshahi M Krack P Litvan I Macias R Bezard E. Initial clinical manifestations of Parkinson's disease: features and pathophysiological mechanisms. Lancet Neurol. (2009) 8:1128–39. 10.1016/S1474-4422(09)70293-5 19909911

[139] Lau A So RWL Lau HHC Sang JC Ruiz-Riquelme A Fleck SC. alpha-Synuclein strains target distinct brain regions and cell types. Nat Neurosci. (2020) 23:21–31. 10.1038/s41593-019-0541-x 31792467 PMC6930851

[140] McColgan P Joubert J Tabrizi SJ Rees G. The human motor cortex microcircuit: insights for neurodegenerative disease. Nat Rev Neurosci. (2020) 21:401–5. 10.1038/s41583-020-0315-1 32555340

[141] Westbrook A van den Bosch R Maatta JI Hofmans L Papadopetraki D Cools R. Dopamine promotes cognitive effort by biasing the benefits versus costs of cognitive work. Science. (2020) 367:1362–6. 10.1126/science.aaz5891 32193325 PMC7430502

[142] Lehericy S Ducros M Van de Moortele PF Francois C Thivard L Poupon C. Diffusion tensor fiber tracking shows distinct corticostriatal circuits in humans. Ann Neurol. (2004) 55:522–9. 10.1002/ana.20030 15048891

[143] Sedrak M Gorgulho A Bari A Behnke E Frew A Gevorkyan I. Diffusion tensor imaging (DTI) and colored fractional anisotropy (FA) mapping of the subthalamic nucleus (STN) and the globus pallidus interna (GPi). Acta Neurochir. (2010) 152:2079–84. 10.1007/s00701-010-0813-4 20890778 PMC2991232

[144] Hauptman JS DeSalles AA Espinoza R Sedrak M Ishida W. Potential surgical targets for deep brain stimulation in treatment-resistant depression. Neurosurg Focus. (2008) 25:E3. 10.3171/FOC/2008/25/7/E3 18590380

[145] Andica C Kamagata K Hatano T Okuzumi A Saito A Nakazawa M. Neurite orientation dispersion and density imaging of the nigrostriatal pathway in Parkinson's disease: retrograde degeneration observed by tract-profile analysis. Parkinsonism Relat Disord. (2018) 51:55–60. 10.1016/j.parkreldis.2018.02.046 29525556

[146] Theisen F Leda R Pozorski V Oh JM Adluru N Wong R. Evaluation of striatonigral connectivity using probabilistic tractography in Parkinson's disease. Neuroimage Clin. (2017) 16:557–63. 10.1016/j.nicl.2017.09.009 28971007 PMC5608174

[147] Menke RA Scholz J Miller KL Deoni S Jbabdi S Matthews PM. MRI characteristics of the substantia nigra in Parkinson's disease: a combined quantitative T1 and DTI study. Neuroimage. (2009) 47:435–41. 10.1016/j.neuroimage.2009.05.017 19447183

[148] Scherfler C Schocke MF Seppi K Esterhammer R Brenneis C Jaschke W. Voxel-wise analysis of diffusion weighted imaging reveals disruption of the olfactory tract in Parkinson's disease. Brain. (2006) 129:538–42. 10.1093/brain/awh674 16272163

[149] Mentzel TQ Lieverse R Levens A Mentzel CL Tenback DE Bakker PR. Reliability and validity of an instrument for the assessment of bradykinesia. Psychiatr Res. (2016) 238:189–95. 10.1016/j.psychres.2016.02.011 27086232

[150] Yao N Cheung C Pang S Shek-kwan Chang R Lau KK Suckling J. Multimodal MRI of the hippocampus in Parkinson's disease with visual hallucinations. Brain Struct Funct. (2016) 221:287–300. 10.1007/s00429-014-0907-5 25287513 PMC4720723

[151] Buchert R Lange C Spehl TS Apostolova I Frings L Jonsson C. Diagnostic performance of the specific uptake size index for semi-quantitative analysis of I-123-FP-CIT SPECT. Harmonized multi-center research setting versus typical clinical single-camera setting. EJNMMI Res. (2019) 9:37. 10.1186/s13550-019-0506-9 31065816 PMC6505020

[152] Jakobson Mo S Axelsson J Jonasson L Larsson A Ogren MJ Ogren M. Dopamine transporter imaging with [(18)F]FE-PE2I PET and [(123)I]FP-CIT SPECT-a clinical comparison. EJNMMI Res. (2018) 8:100. 10.1186/s13550-018-0450-0 30443684 PMC6238014

[153] Prashanth R Roy SD Mandal PK Ghosh S. High-accuracy classification of Parkinson's disease through shape analysis and surface fitting in 123I-Ioflupane SPECT imaging. IEEE J Biomed Health Inform. (2017) 21:794–802. 10.1109/JBHI.2016.2547901 28113827

[154] Augimeri A Cherubini A Cascini GL Galea D Caligiuri ME Barbagallo G. CADA-computer-aided DaTSCAN analysis. EJNMMI Phys. (2016) 3:4. 10.1186/s40658-016-0140-9 26879864 PMC4754234

[155] Kraemmer J Kovacs GG Perju-Dumbrava L Pirker S Traub-Weidinger T Pirker W. Correlation of striatal dopamine transporter imaging with post mortem substantia nigra cell counts. Mov Disord. (2014) 29:1767–73. 10.1002/mds.25975 25048738

[156] Walker Z Jaros E Walker RW Lee L Costa DC Livingston G. Dementia with Lewy bodies: a comparison of clinical diagnosis, FP-CIT single photon emission computed tomography imaging and autopsy. J Neurol Neurosurg Psychiatr. (2007) 78:1176–81. 10.1136/jnnp.2006.110122 17353255 PMC2117602

[157] Yang J Archer DB Burciu RG Muller M Roy A Ofori E. Multimodal dopaminergic and free-water imaging in Parkinson's disease. Parkinsonism Relat Disord. (2019) 62:10–5. 10.1016/j.parkreldis.2019.01.007 30639168 PMC6589363

[158] Zhang Y Wu IW Tosun D Foster E Schuff N Parkinson's Progression Markers I. Progression of regional microstructural degeneration in Parkinson's disease: a multicenter diffusion tensor imaging study. PLoS ONE. (2016) 11:e0165540. 10.1371/journal.pone.0165540 27798653 PMC5087900

[159] Lenfeldt N Eriksson J Astrom B Forsgren L Mo SJ. Fractional anisotropy and mean diffusion as measures of dopaminergic function in parkinson's disease: challenging results. J Parkinsons Dis. (2017) 7:129–42. 10.3233/JPD-161011 28106567

[160] Lorio S Sambataro F Bertolino A Draganski B Dukart J. The combination of DAT-SPECT, structural and diffusion MRI predicts clinical progression in Parkinson's disease. Front Aging Neurosci. (2019) 11:57. 10.3389/fnagi.2019.00057 30930768 PMC6428714

[161] Ofori E Pasternak O Planetta PJ Li H Burciu RG Snyder AF. Longitudinal changes in free-water within the substantia nigra of Parkinson's disease. Brain. (2015) 138:2322–31. 10.1093/brain/awv136 25981960 PMC4840947

[162] Burciu RG Ofori E Archer DB Wu SS Pasternak O McFarland NR. Progression marker of Parkinson's disease: a 4-year multi-site imaging study. Brain. (2017) 140:2183–92. 10.1093/brain/awx146 28899020 PMC6057495

[163] Pozorski V Oh JM Adluru N Merluzzi AP Theisen F Okonkwo O. Longitudinal white matter microstructural change in Parkinson's disease. Hum Brain Mapp. (2018) 39:4150–61. 10.1002/hbm.24239 29952102 PMC6128734

[164] Surova Y Nilsson M Lampinen B Latt J Hall S Widner H. Alteration of putaminal fractional anisotropy in Parkinson's disease: a longitudinal diffusion kurtosis imaging study. Neuroradiology. (2018) 60:247–54. 10.1007/s00234-017-1971-3 29368035 PMC5799343

[165] Miyasaki JM Martin W Suchowersky O Weiner WJ Lang AE Practice parameter: initiation of treatment for Parkinson's disease: an evidence-based review: report of the quality standards subcommittee of the American academy of neurology. Neurology. (2002) 58:11–7. 10.1212/WNL.58.1.11 11781398

[166] Elm JJ Goetz CG Ravina B Shannon K Wooten GF Tanner CM. A responsive outcome for Parkinson's disease neuroprotection futility studies. Ann Neurol. (2005) 57:197–203. 10.1002/ana.20361 15668964

[167] Ravina B Marek K Eberly S Oakes D Kurlan R Ascherio A. Dopamine transporter imaging is associated with long-term outcomes in Parkinson's disease. Mov Disord. (2012) 27:1392–7. 10.1002/mds.25157 22976926 PMC5404810

[168] Simuni T Siderowf A Lasch S Coffey CS Caspell-Garcia C Jennings D. longitudinal change of clinical and biological measures in early Parkinson's disease: Parkinson's progression markers initiative cohort. Mov Disord. (2018) 33:771–82. 10.1002/mds.27361 29572948 PMC6001458

[169] Mishra VR Sreenivasan KR Zhuang X Yang Z Cordes D Walsh RR. Influence of analytic techniques on comparing DTI-derived measurements in early stage Parkinson's disease. Heliyon. (2019) 5:e01481. 10.1016/j.heliyon.2019.e01481 31008407 PMC6458486

[170] Ito M Watanabe H Kawai Y Atsuta N Tanaka F Naganawa S. Usefulness of combined fractional anisotropy and apparent diffusion coefficient values for detection of involvement in multiple system atrophy. J Neurol Neurosurg Psychiatr. (2007) 78:722–8. 10.1136/jnnp.2006.104075 17353258 PMC2117692

[171] Wang PS Wu HM Lin CP Soong BW. Use of diffusion tensor imaging to identify similarities and differences between cerebellar and Parkinsonism forms of multiple system atrophy. Neuroradiology. (2011) 53:471–81. 10.1007/s00234-010-0757-7 20737142

[172] Ota M Nakata Y Ito K Kamiya K Ogawa M Murata M. Differential diagnosis tool for parkinsonian syndrome using multiple structural brain measures. Comput Math Methods Med. (2013) 2013:571289. 10.1155/2013/571289 23573171 PMC3615618

[173] Peran P Barbagallo G Nemmi F Sierra M Galitzky M Traon AP. MRI supervised and unsupervised classification of Parkinson's disease and multiple system atrophy. Mov Disord. (2018) 33:600–8. 10.1002/mds.27307 29473662

[174] Du G Lewis MM Kanekar S Sterling NW He L Kong L. Combined diffusion tensor imaging and apparent transverse relaxation rate differentiate Parkinson disease and atypical Parkinsonism. AJNR Am J Neuroradiol. (2017) 38:966–72. 10.3174/ajnr.A5136 28364007 PMC5433885

[175] Umemura A Oeda T Hayashi R Tomita S Kohsaka M Yamamoto K. Diagnostic accuracy of apparent diffusion coefficient and 123I-metaiodobenzylguanidine for differentiation of multiple system atrophy and Parkinson's disease. PLoS ONE. (2013) 8:e61066. 10.1371/journal.pone.0061066 23613784 PMC3629185

[176] Prodoehl J Li H Planetta PJ Goetz CG Shannon KM Tangonan R. Diffusion tensor imaging of Parkinson's disease, atypical parkinsonism, and essential tremor. Mov Disord. (2013) 28:1816–22. 10.1002/mds.25491 23674400 PMC3748146

[177] Baudrexel S Seifried C Penndorf B Klein JC Middendorp M Steinmetz H. The value of putaminal diffusion imaging versus 18-fluorodeoxyglucose positron emission tomography for the differential diagnosis of the Parkinson variant of multiple system atrophy. Mov Disord. (2014) 29:380–7. 10.1002/mds.25749 24243813

[178] Sako W Abe T Murakami N Miyazaki Y Izumi Y Harada M. Imaging-based differential diagnosis between multiple system atrophy and Parkinson's disease. J Neurol Sci. (2016) 368:104–8. 10.1016/j.jns.2016.06.061 27538610

[179] Dickson DW Hauw JJ Agid Y Litvan I Progressive Supranuclear Palsy and Corticobasal Degeneration. 2nd ed. Chichester: Wiley-Blackwell (2011). 10.1002/9781444341256.ch15

[180] Whitwell JL Hoglinger GU Antonini A Bordelon Y Boxer AL Colosimo C. Radiological biomarkers for diagnosis in PSP. Where are we and where do we need to be? Mov Disord. (2017) 32:955–71. 10.1002/mds.27038 28500751 PMC5511762

[181] Blain CR Barker GJ Jarosz JM Coyle NA Landau S Brown RG. Measuring brain stem and cerebellar damage in parkinsonian syndromes using diffusion tensor MRI. Neurology. (2006) 67:2199–205. 10.1212/01.wnl.0000249307.59950.f8 17190944

[182] Worker A Blain C Jarosz J Chaudhuri KR Barker GJ Williams SC. Diffusion tensor imaging of Parkinson's disease, multiple system atrophy and progressive supranuclear palsy: a tract-based spatial statistics study. PLoS ONE. (2014) 9:e112638. 10.1371/journal.pone.0112638 25405990 PMC4236070

[183] Seki M Seppi K Mueller C Potrusil T Goebel G Reiter E. Diagnostic potential of dentatorubrothalamic tract analysis in progressive supranuclear palsy. Parkins Relat Disord. (2018) 49:81–7. 10.1016/j.parkreldis.2018.02.004 29463454

[184] Cherubini A Morelli M Nistico R Salsone M Arabia G Vasta R. Magnetic resonance support vector machine discriminates between Parkinson disease and progressive supranuclear palsy. Mov Disord. (2014) 29:266–9. 10.1002/mds.25737 24323617

[185] Zhang Y Walter R Ng P Luong PN Dutt S Heuer H. Progression of microstructural degeneration in progressive supranuclear palsy and corticobasal syndrome: a longitudinal diffusion tensor imaging study. PLoS ONE. (2016) 11:e0157218. 10.1371/journal.pone.0157218 27310132 PMC4911077

[186] Paviour DC Price SL Jahanshahi M Lees AJ Fox NC. Longitudinal MRI in progressive supranuclear palsy and multiple system atrophy: rates and regions of atrophy. Brain. (2006) 129(Pt 4):1040–9. 10.1093/brain/awl021 16455792

[187] Dutt S Binney RJ Heuer HW Luong P Attygalle S Bhatt P. Progression of brain atrophy in PSP and CBS over 6 months and 1 year. Neurology. (2016) 87:2016–25. 10.1212/WNL.0000000000003305 27742814 PMC5109951

[188] Litvan I Goldman JG Troster AI Schmand BA Weintraub D Petersen RC. Diagnostic criteria for mild cognitive impairment in Parkinson's disease: movement disorder society task force guidelines. Mov Disord. (2012) 27:349–56. 10.1002/mds.24893 22275317 PMC3641655

[189] Aarsland D Andersen K Larsen JP Lolk A Kragh-Sorensen P. Prevalence and characteristics of dementia in Parkinson disease: an 8-year prospective study. Arch Neurol. (2003) 60:387–92. 10.1001/archneur.60.3.387 12633150

[190] Hely MA Reid WG Adena MA Halliday GM Morris JG. The sydney multicenter study of Parkinson's disease: the inevitability of dementia at 20 years. Mov Disord. (2008) 23:837–44. 10.1002/mds.21956 18307261

[191] Bertrand JA Bedetti C Postuma RB Monchi O Genier Marchand D Jubault T. Color discrimination deficits in Parkinson's disease are related to cognitive impairment and white-matter alterations. Mov Disord. (2012) 27:1781–8. 10.1002/mds.25272 23147270

[192] Deng B Zhang Y Wang L Peng K Han L Nie K. Diffusion tensor imaging reveals white matter changes associated with cognitive status in patients with Parkinson's disease. Am J Alzheimers Dis Other Demen. (2013) 28:154–64. 10.1177/1533317512470207 23271331 PMC10852784

[193] Agosta F Canu E Stefanova E Sarro L Tomic A Spica V. Mild cognitive impairment in Parkinson's disease is associated with a distributed pattern of brain white matter damage. Hum Brain Mapp. (2014) 35:1921–9. 10.1002/hbm.22302 23843285 PMC6869219

[194] Matsui H Nishinaka K Oda M Niikawa H Kubori T Udaka F. Dementia in Parkinson's disease: diffusion tensor imaging. Acta Neurol Scand. (2007) 116:177–81. 10.1111/j.1600-0404.2007.00838.x 17714331

[195] Kamagata K Motoi Y Abe O Shimoji K Hori M Nakanishi A. White matter alteration of the cingulum in Parkinson disease with and without dementia: evaluation by diffusion tensor tract-specific analysis. AJNR Am J Neuroradiol. (2012) 33:890–5. 10.3174/ajnr.A2860 22241380 PMC7968830

[196] Chondrogiorgi M Astrakas LG Zikou AK Weis L Xydis VG Antonini A. Multifocal alterations of white matter accompany the transition from normal cognition to dementia in Parkinson's disease patients. Brain Imaging Behav. (2019) 13:232–40. 10.1007/s11682-018-9863-7 29629498

[197] Kamagata K Motoi Y Tomiyama H Abe O Ito K Shimoji K. Relationship between cognitive impairment and white-matter alteration in Parkinson's disease with dementia: tract-based spatial statistics and tract-specific analysis. Eur Radiol. (2013) 23:1946–55. 10.1007/s00330-013-2775-4 23404139 PMC3674338

[198] Wiltshire K Concha L Gee M Bouchard T Beaulieu C Camicioli R. Corpus callosum and cingulum tractography in Parkinson's disease. Can J Neurol Sci. (2010) 37:595–600. 10.1017/S0317167100010751 21059504

[199] Schulz J Pagano G Fernandez Bonfante JA Wilson H Politis M. Nucleus basalis of Meynert degeneration precedes and predicts cognitive impairment in Parkinson's disease. Brain. (2018) 141:1501–16. 10.1093/brain/awy072 29701787 PMC6171218

[200] Caspell-Garcia C Simuni T Tosun-Turgut D Wu IW Zhang Y Nalls M. Multiple modality biomarker prediction of cognitive impairment in prospectively followed *de novo* Parkinson disease. PLoS ONE. (2017) 12:e0175674. 10.1371/journal.pone.0175674 28520803 PMC5435130

[201] Silbert LC Kaye J. Neuroimaging and cognition in Parkinson's disease dementia. Brain Pathol. (2010) 20:646–53. 10.1111/j.1750-3639.2009.00368.x 20522090 PMC3327506

[202] Shin NY Shin YS Lee PH Yoon U Han S Kim DJ. Different functional and microstructural changes depending on duration of mild cognitive impairment in Parkinson disease. AJNR Am J Neuroradiol. (2016) 37:897–903. 10.3174/ajnr.A4626 26705323 PMC7960312

[203] Lee JE Park HJ Park B Song SK Sohn YH Lee JD. A comparative analysis of cognitive profiles and white-matter alterations using voxel-based diffusion tensor imaging between patients with Parkinson's disease dementia and dementia with Lewy bodies. J Neurol Neurosurg Psychiatr. (2010) 81:320–6. 10.1136/jnnp.2009.184747 19828477

[204] Novellino F Vasta R Sarica A Chiriaco C Salsone M Morelli M. Relationship between hippocampal subfields and category cued recall in AD and PDD. A multimodal MRI study. Neuroscience. (2018) 371:506–17. 10.1016/j.neuroscience.2017.12.028 29292073

[205] Bergamino M Keeling EG Mishra VR Stokes AM Walsh RR. Assessing white matter pathology in early-stage Parkinson disease using diffusion MRI. A systematic review. Front Neurol. (2020) 11:314. 10.3389/fneur.2020.00314 32477235 PMC7240075

[206] Tuch DS Reese TG Wiegell MR Makris N Belliveau JW Wedeen VJ. High angular resolution diffusion imaging reveals intravoxel white matter fiber heterogeneity. Magn Reson Med. (2002) 48:577–82. 10.1002/mrm.10268 12353272

[207] Wedeen VJ Wang RP Schmahmann JD Benner T Tseng WY Dai G. Diffusion spectrum magnetic resonance imaging (DSI) tractography of crossing fibers. Neuroimage. (2008) 41:1267–77. 10.1016/j.neuroimage.2008.03.036 18495497

[208] Pasternak O Sochen N Gur Y Intrator N Assaf Y. Free water elimination mapping from diffusion MRI. Magn Reson Med. (2009) 62:717–30. 10.1002/mrm.22055 19623619

[209] Guttuso T Jr Bergsland N Hagemeier J Lichter DG Pasternak O. Substantia nigra free water increases longitudinally in Parkinson disease. AJNR Am J Neuroradiol. (2018) 39:479–84. 10.3174/ajnr.A5545 29419398 PMC6070442

[210] Jensen JH Helpern JA Ramani A Lu H Kaczynski K. Diffusional kurtosis imaging: the quantification of non-gaussian water diffusion by means of magnetic resonance imaging. Magn Reson Med. (2005) 53:1432–40. 10.1002/mrm.20508 15906300

[211] Kamagata K Tomiyama H Hatano T Motoi Y Abe O Shimoji K. A preliminary diffusional kurtosis imaging study of Parkinson disease: comparison with conventional diffusion tensor imaging. Neuroradiology. (2014) 56:251–8. 10.1007/s00234-014-1327-1 24468858

[212] Vriend C van den Heuvel OA Berendse HW van der Werf YD Douw L. Global and subnetwork changes of the structural connectome in *de novo* Parkinson's disease. Neuroscience. (2018) 386:295–308. 10.1016/j.neuroscience.2018.06.050 30004009

[213] Hall JM Shine JM Ehgoetz Martens KA Gilat M Broadhouse KM Szeto JYY. Alterations in white matter network topology contribute to freezing of gait in Parkinson's disease. J Neurol. (2018) 265:1353–64. 10.1007/s00415-018-8846-3 29616302

[214] Tinaz S Lauro PM Ghosh P Lungu C Horovitz SG. Changes in functional organization and white matter integrity in the connectome in Parkinson's disease. Neuroimage Clin. (2017) 13:395–404. 10.1016/j.nicl.2016.12.019 28116232 PMC5226806

[215] Galantucci S Agosta F Stefanova E Basaia S van den Heuvel MP Stojkovic T. Structural brain connectome and cognitive impairment in Parkinson disease. Radiology. (2017) 283:515–25. 10.1148/radiol.2016160274 27924721

[216] Nigro S Riccelli R Passamonti L Arabia G Morelli M Nistico R. Characterizing structural neural networks in *de novo* Parkinson disease patients using diffusion tensor imaging. Hum Brain Mapp. (2016) 37:4500–10. 10.1002/hbm.23324 27466157 PMC6867369

[217] Sharman M Valabregue R Perlbarg V Marrakchi-Kacem L Vidailhet M Benali H. Parkinson's disease patients show reduced cortical-subcortical sensorimotor connectivity. Mov Disord. (2013) 28:447–54. 10.1002/mds.25255 23144002

[218] Sterling NW Du G Lewis MM Swavely S Kong L Styner M. Cortical gray and subcortical white matter associations in Parkinson's disease. Neurobiol Aging. (2017) 49:100–8. 10.1016/j.neurobiolaging.2016.09.015 27776262 PMC5154847

[219] Pagonabarraga J Kulisevsky J. Cognitive impairment and dementia in Parkinson's disease. Neurobiol Dis. (2012) 46:590–6. 10.1016/j.nbd.2012.03.029 22484304

[220] Illan-Gala I Montal V Borrego-Ecija S Vilaplana E Pegueroles J Alcolea D. Cortical microstructure in the behavioural variant of frontotemporal dementia: looking beyond atrophy. Brain. (2019) 142:1121–33. 10.1093/brain/awz031 30906945 PMC6439330

[221] Ball G Srinivasan L Aljabar P Counsell SJ Durighel G Hajnal JV. Development of cortical microstructure in the preterm human brain. Proc Natl Acad Sci USA. (2013) 110:9541–6. 10.1073/pnas.1301652110 23696665 PMC3677430

[222] Andica C Kamagata K Hatano T Saito A Uchida W Ogawa T. Free-water imaging in white and gray matter in Parkinson's disease. Cells. (2019) 8:839. 10.3390/cells8080839 31387313 PMC6721691

[223] Weston PS Simpson IJ Ryan NS Ourselin S Fox NC. Diffusion imaging changes in grey matter in Alzheimer's disease: a potential marker of early neurodegeneration. Alzheimers Res Ther. (2015) 7:47. 10.1186/s13195-015-0132-3 26136857 PMC4487800

[224] Sampedro F Perez-Gonzalez R Martinez-Horta S Marin-Lahoz J Pagonabarraga J Kulisevsky J. Serum neurofilament light chain levels reflect cortical neurodegeneration in *de novo* Parkinson's disease. Parkinsonism Relat Disord. (2020) 74:43–9. 10.1016/j.parkreldis.2020.04.009 32334380

[225] Sampedro F Martinez-Horta S Marin-Lahoz J Pagonabarraga J Kulisevsky J. Longitudinal intracortical diffusivity changes in de-novo Parkinson's disease: a promising imaging biomarker. Parkinsonism Relat Disord. (2019) 68:22–5. 10.1016/j.parkreldis.2019.09.031 31621613

[226] Zhang Y Vakhtin AV Jennings JS Massaband P Wintermark M Craig PL. Diffusion tensor tractography of brainstem fibers and its application in pain. PLoS ONE. (2019) 15:e0213952. 10.1371/journal.pone.0213952 32069284 PMC7028272

[227] Tolosa E Wenning G Poewe W The diagnosis of Parkinson's disease. Lancet Neurol. (2006) 5:75–86. 10.1016/S1474-4422(05)70285-4 16361025

