# Corrigendum: Diffusion Tensor Imaging in Parkinson's Disease and Parkinsonian Syndrome: A Systematic Review

**DOI:** 10.3389/fneur.2020.612069

**Published:** 2020-10-29

**Authors:** Yu Zhang, Marc A. Burock

**Affiliations:** ^1^Department of Psychiatry, War Related Illness and Injury Study Center, Veterans Affairs Palo Alto Health Care System, Palo Alto, CA, United States; ^2^Department of Psychiatry, Mainline Health, Bryn Mawr Hospital, Bryn Mawr, PA, United States

**Keywords:** diffusion tensor imaging (DTI), fractional anisotropy (FA), Parkinson's progression marker initiative (PPMI), diffusion tensor tractography (DTT), dopaminergic pathway, substantia nigra (SN), Parkinson's disease (PD)

In the original article, reference 31 was incorrectly written as Haghshomar M, Rahmani F. Cognitive challenge to choose healthier food is reflected in heart rate variability. *Front Neurosci*. (2017) 11:353. doi: 10.3389/fnins.2017.00353 It should be Haghshomar M, Rahmani F, Hadi Aarabi M, Shahjouei S, Sobhani S, Rahmani M. White matter changes correlates of peripheral neuroinflammation in patients with Parkinson's disease. *Neuroscience*. (2019) 403:70–8. doi: 10.1016/j.neuroscience.2017.10.050.

The authors apologize for this error and state that this does not change the scientific conclusions of the article in any way. The original article has been updated.

