# Supplementary material for: Diffusion Tensor Imaging in Parkinson's Disease and Parkinsonian Syndrome: A Systematic Review
Source: Front Neurol. 2020 Sep 25;11:531993. doi: 10.3389/fneur.2020.531993 (PMC7546271; doi:10.3389/fneur.2020.531993)
Supplement: Supplementary file 1 [file Data_Sheet_1.docx]

**Supplement Material to:**

**Diffusion Tensor Imaging in Parkinson’s Disease and Parkinsonian Syndrome: A Systematic Review**

**S1). PPMI Data collection**

All imaging and clinical data were collected from the PPMI [1], which was a 5-year observational, international, multicenter, longitudinal study of early-stage, drug-naive idiopathic patients with PD (so-called de-novo PD), and age-matched healthy control (HC) subjects recruited from multiple clinical sites worldwide. The study was approved by the respective institutional review boards of all participating sites, and all subjects provided written informed consent. All participants were recruited at baseline if they met the following criteria: over 30 years old, PD was diagnosed within the past 2 years, and de novo patients with Hoehn and Yahr (H&Y) [2] stage I or II. Further, motor function was assessed using the Movement Disorder Society-sponsored revision of the Unified PD Rating Scale part three (MDS-UPDRS3) [3] at each visit when patients were off-medication for 24 hours. Global cognitive function was assessed using the Montreal Cognitive Assessment (MoCA) [4]. Demographically comparable HC subjects, free of neurological and radiological disorders, were also included. Therefore, the PPMI samples are characterized by newly diagnosed, early-stage, de-novo cases, and involve a large number of young-onset patients.

This study initially included baseline and up to 4-years of annual magnetic resonance imaging (MRI) scans of PD and HC subjects who participated in the PPMI between 2011 and 2017. The exclusion criteria were: (1) clinically diagnosed PD with no dopaminergic deficit, also known as scans without evidence of dopaminergic deficit (SWEDD), (2) MRI qualities failed due to scan artifacts, (3) radiological abnormalities including asymptomatic infarction, hydrocephalus, and benign tumors. Finally, a total of 134 de-novo PD patients with 482 serial MRI data sets (0 to 4 years), and 75 age-matched HC subjects with 146 serial MRI scans were included (**Table S1**).

To analyze DTI changes along with early disease courses, baseline and follow-up groups were redefined based on disease duration after onset, rather than the MRI visit time (See Supplementary Table S1**)**. Specifically, for PD patients, baseline group (PD(Y0)) was determined based on the exam time when PD was diagnosed within the first year (0-12 months). The first-year group (PD(Y1)) was determined based on the MRI time of patients with a PD duration of 1 to 2 years (13-24 months). The second-year group (PD(Y2)) was obtained from the scan time of patients who were diagnosed with PD in the previous 2 to 3 years (25-36 months). MRI data of patients with PD for longer than 3 years (37-79 months) was defined as PD(Y3). Because the PPMI samples involve a large number of young-onset patients, DTI data was analyzed separately for the young-onset group (YPD, age of onset ≤ 50 years, according to the American Parkinson’s Disease Association) and the typical-onset group (OPD, age of onset > 50 years). All HC subjects were split into age-matched YHC (age at MRI ≤ 50 years) and OHC (age at MRI > 50 years) groups.

**S2) Image acquisition and processing**

MRI of all participants was performed at 11 PPMI sites using a standardized protocol with consistent scan sequences and parameters on a 3 Tesla MRI system (all Siemens Healthcare). Two MRI scan types were collected for each subject at each annual time point: (1) three-dimensional magnetization prepared rapid gradient echo (MPRAGE) sequence for structural T1-weighted anatomical imaging (T1WI, TR/TR/TI = 2300/3/900ms; 1 mm isotropic resolution; twofold acceleration; sagittal-oblique angulation), and (2) two-dimensional single-shot echo-planar sequence for DTI (TR ranged from 8,400 to 8,800 depending on subjects’ heart rate, TE = 88ms, 2 mm isotropic resolution; 72 contiguous slices, twofold acceleration, axial-oblique aligned along the anterior-posterior commissure, with diffusion-weighted gradients along 64 sensitization directions and a b factor of 0, 1000s/mm^2^).

Images were processed at the laboratory (Center for Imaging of Neurodegenerative Diseases at the University of California, San Francisco) by the lead author (Y.Z.) in 2017, using an automated quality and processing pipeline developed in-house [5]. The automated, longitudinal image processing was performed as follows: (1) The raw diffusion-weighted images were first corrected for eddy currents and head motion, followed by reconstruction of the conventional DTI scalar parameter maps, such as FA and MD maps. (2) FA maps of each individual were registered onto their structural T1WI using an affine registration algorithm to reduce distortions of DTI relative to non-distorted T1WI. (3) After correcting for distortion, intra-subject affine registration was performed to reduce measurement bias. In brief, baseline and all follow-up FA maps of each subject were registered onto their subject-specific time-averaged template. The template was created by averaging the FA maps at all time points that were co-registered via a rigid body transformation algorithm to a population-based FA template in Montreal Neurological Institute (MNI) space [6]. (4) Next, an inter-subject registration was performed for the group analyses, using a nonlinear deformation algorithm, to transform each subject-specific FA template into the standard MNI space. Finally, the nonlinear transformation parameter for each subject was applied to the baseline and follow-up FA maps after intra-subject registration to ensure voxel-based analyses for all longitudinal data in the standard MNI space.

All above registration steps were performed using according functions (rigid body, affine, deformation algorithms) in ANTs [7]. Transformation parameters of registration steps from the FA map were recorded and also applied on to MD.

**S3) Voxel-based analyses and key ROIs extraction**

After registering FA maps of all subjects and time points to MNI space, the resulting images were smoothed using a full-width half-maximum (FWHM) of 4 mm, to reduce the effects of local misregistration. Using SPM12 (<https://www.fil.ion.ucl.ac.uk/spm/software/spm12>) implemented in Matlab 2016a on a Linux operator. Pairwise group comparisons were performed as the following: YPD(Y0) (N=29) *vs.* overall YHC (N=30); YPD(Y3) (N=19) *vs.* overall YHC (N=30); OPD(Y0) (N=104) *vs.* overall OHC (N=116); YPD(Y3) (N=72) *vs.* overall YHC (N=116). FA values greater than 0.20 were applied to restrict FA results to predominantly white matter. The two-tailed statistical significance for main effects (group differences) was set to an uncorrected voxel-level *p-*value of 0.001, displaying clusters larger than 20 voxels.

To improve reliability and validity of the findings resulted from voxel-based analyses, we further measured DTI variables in two key ROIs – the substantia nigra (SN) and the corticospinal tract (CST), which are the key anatomy that are relevant to PD pathology. These ROIs were determined to be the clusters in the key anatomy that presented with the maximal group differences above the default *p* threshold (uncorrected *p-*value=0.001) on the voxel-based analyses. Once these specific clusters were determined, they were transferred to binary masks to be the key ROIs in MNI space. Mean FA and MD values of the voxels in the key ROIs were extracted from the unsmoothed FA and MD images that were priorly registered into the MNI space. Furthermore, the values of the bilateral ROIs were averaged.

**Supplementary Table S1.** Demographic and clinical characteristics of the PPMI DTI data.

| **Duration** | **Y0-1** | **Y1-2** | **Y2-3** | **Y>3** |
| --- | --- | --- | --- | --- |
| YPD (Number) | 29 | 27 | 22 | 19 |
| Age at MRI (Mean (SD)) | 46.1 (4.2) | 46.9 (4.2) | 49.2 (3.7) | 50.6 (3.7) |
| Gender (Male%) | 66 | 70 | 68 | 58 |
| H&Y grade (Mean (SD)) | 1.6 (0.5) | 1.7 (0.4) | 1.8 (0.4) | 1.8 (0.4) |
| MDS-UPDRS3 (Mean (SD)) | 18.0 (7.8) | 19.8 (7.5) | 24.6 (8.2) | 25.2 (11.5) |
| MoCA (Mean (SD)) | 28.9 (1.3) | 28.4 (1.5) | 28.7 (1.2) | 28.5 (1.6) |
| Medication (Medicated%) | 0 | 66.7 | 86.4 | 89.5 |
| YHC (Number) | 15 | 9 | 4 | 2 |
| Age at MRI (Mean (SD)) | 42.3 (5.5) | 43.4 (5.8) | 43.8 (8.9) | 49 (1.4) |
| Gender (Male%) | 73 | 55 | 50 | 100 |
| MDS-UPDRS3 (Mean (SD)) | 0.2 (0.4) | 0.7 (1.3) | 0.5 (1.0) | 2.0 (2.8) |
| MoCA (Mean (SD)) | 29.0 (1.1) | 27.9 (2.3) | 28.5 (1.0) | 28 (0) |
| OPD (Number) | 104 | 107 | 102 | 72 |
| Age at MRI (Mean (SD)) | 63.8 (7.0) | 64.0 (6.8) | 65.6 (6.9) | 67.2 (6.5) |
| Gender (Male%) | 62 | 67 | 64 | 75 |
| H&Y grade (Mean (SD)) | 1.6 (0.5) | 1.8 (0.5) | 1.8 (0.5) | 1.9 (0.6) |
| MDS-UPDRS3 (Mean (SD)) | 20.5 (8.6) | 24.3 (11.7) | 25.8 (13.2) | 29.1 (11.6) |
| MoCA (Mean (SD)) | 27.4 (2.2) | 26.5 (2.7) | 26.2 (3.1) | 26.3 (3.0) |
| Medication (Medicated%) | 0 | 61.7 | 79.4 | 90.3 |
| OHC (Number) | 60 | 47 | 6 | 3 |
| Age at MRI (Mean (SD)) | 63.4 (7.6) | 65.2 (7.0) | 63.8 (8.8) | 66 (12) |
| Gender (Male%) | 63 | 64 | 83 | 100 |
| MDS-UPDRS3 (Mean (SD)) | 0.7 (1.5) | 1.0 (2.0) | 0.8 (1.0) | 0 (0) |
| MoCA (Mean (SD)) | 28.1 (1.1) | 27.2 (2.0) | 28.5 (0.8) | 26.7 (3.1) |
| Total PD (Number) | 133 | 134 | 124 | 91 |
| Total HC (Number) | 75 | 56 | 10 | 5 |

**References**

1. Marek K: **The Parkinson Progression Marker Initiative (PPMI)**. *Prog Neurobiol* 2011, **95**(4):629-635.

2. Hoehn MM, Yahr MD: **Parkinsonism: onset, progression, and mortality. 1967**. *Neurology* 2001, **57**(10 Suppl 3):S11-26.

3. Goetz CG, Fahn S, Martinez-Martin P, Poewe W, Sampaio C, Stebbins GT, Stern MB, Tilley BC, Dodel R, Dubois B *et al*: **Movement Disorder Society-sponsored revision of the Unified Parkinson's Disease Rating Scale (MDS-UPDRS): Process, format, and clinimetric testing plan**. *Mov Disord* 2007, **22**(1):41-47.

4. Paulus W, Jellinger K: **The neuropathologic basis of different clinical subgroups of Parkinson's disease**. *J Neuropathol Exp Neurol* 1991, **50**(6):743-755.

5. Schuff N, Wu IW, Buckley S, Foster ED, Coffey CS, Gitelman DR, Mendick S, Seibyl J, Simuni T, Zhang Y *et al*: **Diffusion imaging of nigral alterations in early Parkinson's disease with dopaminergic deficits**. *Mov Disord* 2015, **30**(14):1885-1892.

6. Oishi K, Faria A, Jiang H, Li X, Akhter K, Zhang J, Hsu JT, Miller MI, van Zijl PC, Albert M *et al*: **Atlas-based whole brain white matter analysis using large deformation diffeomorphic metric mapping: application to normal elderly and Alzheimer's disease participants**. *Neuroimage* 2009, **46**(2):486-499.

7. Avants BB, Tustison NJ, Song G, Cook PA, Klein A, Gee JC: **A reproducible evaluation of ANTs similarity metric performance in brain image registration**. *Neuroimage* 2011, **54**(3):2033-2044.

8. Benjamini Y, Hochberg Y: **Controlling the false discovery rate: a practical and powerful approach to multiple testing**. *Journal of the Royal Statistical Society* 1995, **Series B. 57**(1):289-300.
